# Exon-4 Mutations in KRAS Affect MEK/ERK and PI3K/AKT Signaling in Human Multiple Myeloma Cell Lines

**DOI:** 10.3390/cancers12020455

**Published:** 2020-02-16

**Authors:** Susann Weißbach, Sofia Catalina Heredia-Guerrero, Stefanie Barnsteiner, Lukas Großhans, Jochen Bodem, Hanna Starz, Christian Langer, Silke Appenzeller, Stefan Knop, Torsten Steinbrunn, Simone Rost, Hermann Einsele, Ralf Christian Bargou, Andreas Rosenwald, Thorsten Stühmer, Ellen Leich

**Affiliations:** 1 Institute of Pathology, University of Würzburg, Josef-Schneider-Str. 2, 97080 Würzburg, Germany; susi.weissbach@googlemail.com (S.W.); sofia.heredia_guerrero@uni-wuerzburg.de (S.C.H.-G.); barnsteiner.stefanie@gmail.com (S.B.); hannastarz@web.de (H.S.); rosenwald@uni-wuerzburg.de (A.R.); 2 Comprehensive Cancer Center Mainfranken, University Hospital of Würzburg, Josef-Schneider-Str. 6, 97080 Würzburg, Germany; lukas.grosshans@stud-mail.uni-wuerzburg.de (L.G.); silke.appenzeller@uni-wuerzburg.de (S.A.); bargou_r@ukw.de (R.C.B.); 3 Institute of Virology and Immunobiology, University of Würzburg, Versbacher Str. 7, 97078 Würzburg, Germany; jochen.bodem@vim.uni-wuerzburg.de; 4 Department of Internal Medicine III, University Hospital of Ulm, Albert-Einstein-Allee 23, 89081 Ulm, Germany; christian.langer@klinikum-kempten.de; 5 Department of Internal Medicine II, University Hospital Würzburg, Oberdürrbacher Str. 6, 97080 Würzburg, Germany; knop_s@ukw.de (S.K.); steinbrunn_t@ukw.de (T.S.); einsele_h@ukw.de (H.E.); 6 Institute of Human Genetics, Biocenter, University of Würzburg, Am Hubland, 97074 Würzburg, Germany; simone.rost@biozentrum.uni-wuerzburg.de

**Keywords:** multiple myeloma, KRAS, MEK/ERK-signaling, AKT-signaling, amplicon sequencing

## Abstract

Approximately 20% of multiple myeloma (MM) cases harbor a point mutation in *KRAS*. However, there is still no final consent on whether *KRAS*-mutations are associated with disease outcome. Specifically, no data exist on whether *KRAS*-mutations have an impact on survival of MM patients at diagnosis in the era of novel agents. Direct blockade of KRAS for therapeutic purposes is mostly impossible, but recently a mutation-specific covalent inhibitor targeting KRAS^p.G12C^ entered into clinical trials. However, other *KRAS* hotspot-mutations exist in MM patients, including the less common exon-4 mutations. For the current study, the coding regions of *KRAS* were deep-sequenced in 80 newly diagnosed MM patients, uniformely treated with three cycles of bortezomib plus dexamethasone and cyclophosphamide (VCD)-induction, followed by high-dose chemotherapy and autologous stem cell transplantation. Moreover, the functional impact of KRAS^p.G12A^ and the exon-4 mutations p.A146T and p.A146V on different survival pathways was investigated. Specifically, KRAS^WT^, KRAS^p.G12A^, KRAS^p.A146T^, and KRAS^p.A146V^ were overexpressed in HEK293 cells and the KRAS^WT^ MM cell lines JJN3 and OPM2 using lentiviral transduction and the Sleeping Beauty vector system. Even though *KRAS*-mutations were not correlated with survival, all KRAS-mutants were found capable of potentially activating MEK/ERK- and sustaining PI3K/AKT-signaling in MM cells.

## 1. Introduction

Oncogenic activation of RAS-dependent pathways is a hallmark in a wide range of solid and hematological malignancies [1,2], including multiple myeloma (MM) [3,4]. In their GTP-bound state, RAS proteins show a high affinity towards downstream effectors such as PI3K, RAF, RAL, RIN, TIAM, and PLCε, leading to the activation of the mitogen-activated protein/extracellular signal-regulated kinase kinase 1 and 2 (MEK1/2) extracellular signal-regulated kinase 1 and 2 (ERK1/2) module, the PI3K/AKT pathway, and other signaling cascades [5,6,7,8]. Thus, RAS proteins are involved in the regulation of proliferation and survival. Up to 40% of MM cases harbor a point mutation in either *KRAS* (~20%) or *NRAS* (~20%) and it has been shown that survival of MM cell lines depends on oncogenic RAS [4,9,10]. Given the important role of mutated KRAS for the development and progression of many tumor entities, targeting this oncogenic driver addresses an urgent clinical need. However, mutated KRAS does not possess an accessible active site to which small molecules could bind [1]. Targeting KRAS directly is thus a great challenge and after more than three decades of research, KRAS-inhibitors have still not been implemented in cancer treatment [1,11]. However, recently AMG 510—a covalently binding inhibitor of the p.G12C mutant of KRAS—was developed by leveraging the H95/Y96/Q99 cryptic pocket in GDP-KRASG12C, and has entered a phase 1/2 clinical trial (NCT03600883) after biopharmaceutical optimization [1]. The prognostic outcome of patients with *RAS* mutated MM has been assessed in several studies with contradicting conclusions, which may at least in part reflect the fact that different treatment regimens have been used [3,12,13,14,15,16,17]. Of note, in trials treating relapsed/refractory patients with proteasome inhibitors, no significant difference in overall survival between *RAS*-mutant and *RAS*-WT patients was found [4,18].

Oncogenic point mutations in *RAS* most commonly occur in codons 12 and 13 of exon-2 and in codon 61 of exon-3 [9,19,20,21,22]. These mutations impair intrinsic GTPase activity, thus preventing RAS deactivation [8]. Consequently, RAS remains constitutively active and promotes cancer cell growth and survival [2]. Moreover, mutations in *KRAS* are found in exon-4 (p.A146, p.K117) in approximately 4% of primary colorectal cancers and in 10% of colorectal cancer cell lines [23,24], as well as in a few MM patients and in the MM cell line AMO1 [9,19,20,22,25,26]. Exon-4 mutations at codon 146 affect an evolutionarily conserved region which is predicted to interact with the guanine base of GDP. These lesions do not impair intrinsic KRAS GTPase activity [24,27], but increase the rate of guanine nucleotide exchange, thus resulting in increased net-activation [28]. However, the activating potential of increased nucleotide exchange was deemed to be lower than that of decreased GTPase activity, because the latter translated into superior capacity for transformation [28]. Nevertheless, in vitro and in vivo investigations with colorectal cancer models showed that exon-4 mutations conferred a dependence on MEK/ERK-signaling and resistance to EGFR-targeted agents. They were also accompanied by conversion to homozygosity and copy number (CN) gains of *KRAS* which may augment the activity of mutations at this site [24]. However, the functional investigations were specifically focused on a single mutation located in exon-4 (p.A146T) and on the exon-2 mutation p.G12D, and they were limited to MEK/ERK-signaling and effects of MEK/ERK- and EGFR-inhibitors [24]. Moreover, to our knowledge, no data about the functional role of exon-4 mutations in MM are available.

To investigate if the occurrence of *KRAS*-mutations has a prognostic role for newly diagnosed MM patients treated with current treatment regimens including novel agents, and whether the exon-4 mutations may play a functional role in MM pathogenesis, we performed deep-sequencing of the coding regions of *KRAS* in samples from 80 MM patients at diagnosis, who were then uniformly treated with bortezomib and high-dose chemotherapy. Stable overexpression cell line models were used to functionally investigate the impact of the exon-2 mutant KRAS^p.G12A^ and the exon-4 mutants KRAS^p.A146T^ and KRAS^p.A146V^ on different survival pathways in MM and non-MM cell lines.

## 2. Results

### 2.1. Sequencing, Filtering, and Validation

The sequencing of *KRAS* in newly diagnosed MM (NDMM) samples from 80 patients of the “Deutsche Studiengruppe Multiples Myelom” (DSMM) uniformly treated with three cycles of bortezomib plus dexamethasone and cyclophosphamide (VCD) and subsequent stem cell mobilization, high-dose chemotherapy, and autologous stem cell transplantation and 12 MM cell lines revealed a median on-target coverage of 121 with 92–140 reads per sample. A few samples showed only little or no coverage in exon 3 and were thus re-sequenced using Sanger sequencing. In total, 104 base substitutions or indels were detected and 34 substitutions and nine indels were assigned to the coding region of *KRAS*. The indels predominantly occurred in homopolymers and were thus completely excluded from further analysis. In the next filtering steps, synonymous single nucleotide variations (SNVs), non-synonymous SNVs with a DNA variant allele frequency (VAF) of <5%, and SNVs that occurred in only the forward or the reverse reads were excluded, which finally revealed 12 non-synonymous SNVs in 24 primary MM and three MM cell lines for validation with Sanger sequencing or high resolution melting (HRM). Six SNVs could not be confirmed by Sanger sequencing or HRM or were present in both the tumor and corresponding normal samples, and for one SNV no suitable HRM primers could be designed. The remaining six SNVs that were detected in 16 patients could be confirmed by Sanger sequencing or by HRM. Two MM cell lines (MM1.S and RPMI8226) carried SNVs that were also detected in a primary MM sample and one additional SNV was detected in the MM cell line AMO1, as described previously [26].

### 2.2. Exon-4 KRAS-Mutations Are Rare in the Current Study Cohort

**Figure 1:**
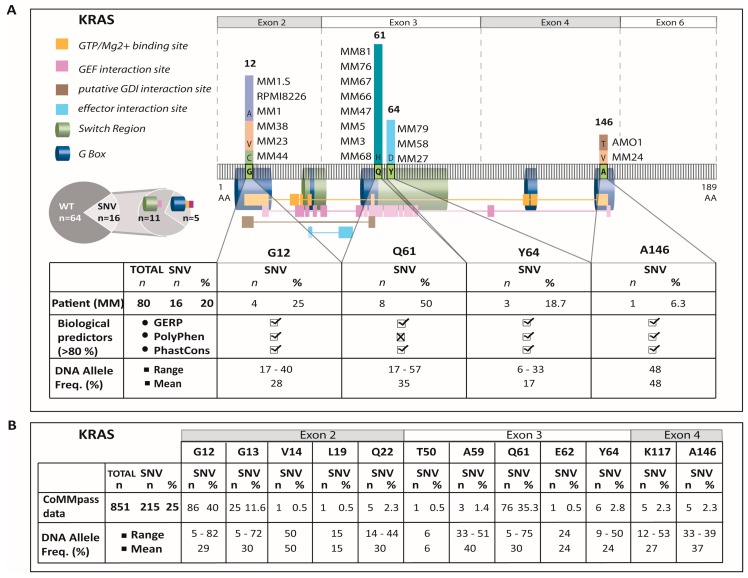
Distribution of *KRAS* mutations in the MM cohort studied, and also including two MM cell lines (AMO1, MM1.S) with known *KRAS*-mutations [26], (**A**) and within the CoMMpass dataset which was generated as part of the Multiple Myeloma Research Foundation Personalized Medicine Initiatives (Initiatives 2014) [25]; (**B**). Additionally shown is the biological relevance of the *KRAS*-mutations detected in the current cohort according to the bioinformatic predictor tools GERP, PolyPhen and PhastCons. (**A**) The variant allele frequency is given as an approximate indicator of the level of clonality within the sample (**A**,**B**). The A146V mutation which was detected in a MM patient of the DSMM cohort was assigned to the group of clonal KRAS-mutations (see Figure S1). *n*: number, MM: multiple myeloma, AA: amino acid, SNV: single nucleotide variant.

*KRAS*-mutations were detected in 20% (16/80) of NDMM patients. The mutations accumulated in hotspot regions of exons 2, 3, and 4 at positions p.G12, p.Q61, p.Y64, and p.A146 (Figure 1A) which is similar to the distribution that was revealed from the CoMMpass dataset (Figure 1B) and the distributions reported by others [9,19,20,22,25]. The most commonly affected site in the current cohort was p.Q61, while only one patient was affected by an exon-4 mutation. According to the calculated tumor population rate, which was determined using the VAF and the copy number (CN)-status for *KRAS*, the cohort was divided into nine patients with clonal *KRAS*-mutations and seven patients with subclonal *KRAS*-mutations at diagnosis (Figure S1). The patient with the exon-4 mutation p.A146V showed a VAF of 48% and was assigned to the group with clonal *KRAS*-mutations in the current cohort (Figure S1). Likewise, the high VAF numbers at DNA level for p.A146 mutations in the CoMMpass dataset (33–39% (*n* = 5); Figure 1B) suggested a clonal or at least major subclonal presence. This is further underscored by RNA-level VAF-analyses also provided by the CoMMpass database, which for p146 mutations range between 47–54% (*n* = 4) [25].

### 2.3. KRAS-Mutations at Diagnosis Have No Predictive Value in Patients Treated with VCD and High-Dose Chemotherapy

In the current dataset, the occurrence of *KRAS*-mutations (20%) neither significantly correlated with cytogenetic alterations such as t(4;14), t(11;14) or t(14;16), nor with deletions in 13q or 17p (Table 1). The co-occurrence of *KRAS*- and *DIS3*-mutations was observed in just one patient. Thus, in contrast to a previous analysis [29], no significant association between the occurrence of *KRAS*- and *DIS3*-mutations was found in the current patient cohort (Table 1, *p* = 0.676).

**Figure 2:**
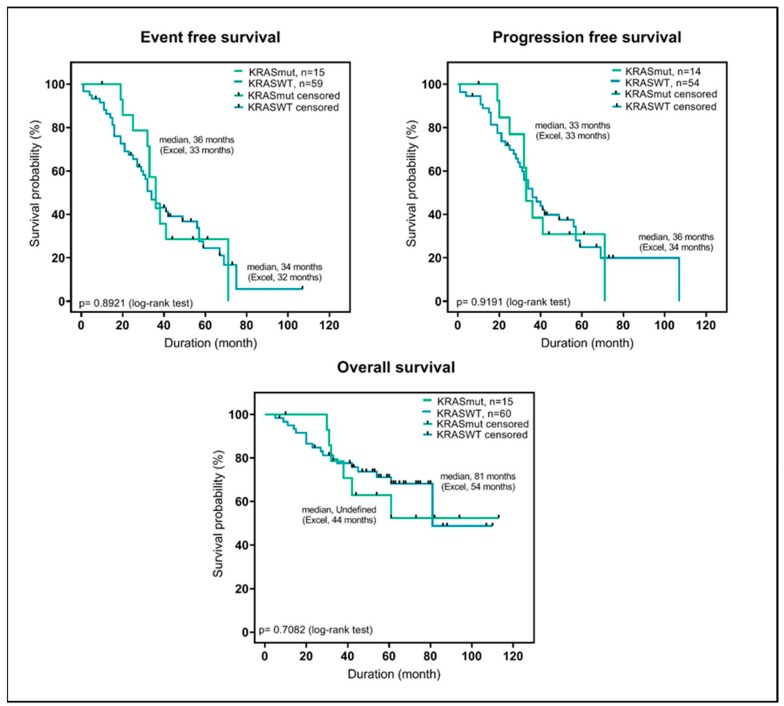
Kaplan Meier plots, showing the difference in overall survival, event free survival, and progression free survival between MM cases with or without *KRAS* mutation.

Moreover, *KRAS*-mutations were not significantly associated with clinical parameters such as age or stage of the disease (Table S1) and did not correlate with outcome (progression free survival, event free survival, overall survival) or with response to therapy (Table S1, Figure 2).

The separation into clonal or subclonal presence of *KRAS*-mutations revealed a trend toward reduced overall survival in patients with clonal *KRAS*-mutations versus patients with minor subclone *KRAS*-mutations (43 months vs. 63 months, *p* = n.s.). However, these differences did not reach statistical significance (Figure S1). Likewise, neither clonal nor subclonal *KRAS*-mutations were associated with the occurrence of classic cytogenetic alterations (Figure S1).

### 2.4. Exon-4 Mutation in AMO1 Cells is Accompanied by Increased CN-Stage and Gene Expression Levels

To investigate whether mutations in *KRAS* correlate with CN-alterations, copy neutral loss of heterozygosity, or differences in gene expression, SNP6.0 and HG-U133 plus 2.0 microarrays were used to interrogate the six MM cell lines AMO1, U266, MM1.S, OPM2, JJN3, and L363, which have previously been analyzed by whole exome sequencing [26] and were included in the current amplicon sequencing approach. Interestingly, a CN gain in 12p12.1-12q11 also affecting *KRAS* (CN-state 4) was observed in AMO1 cells, which were found to have an allele frequency of 46% for KRAS^p.A146T^. The KRAS^WT^-cell line OPM2 showed a gain in 12p12.1 involving also *KRAS* with a CN-state of 3. In contrast, no CN gain was observed for KRAS^p.G12A^-mutant MM1.S (~46% allele frequency) and KRAS^WT^ L363, U266, and JJN3 cells. Consistent with the CN gain in AMO1 and OPM2, KRAS-expression was increased only in these two cell lines (2.3-fold and 1.1-fold higher than the median expression, respectively).

### 2.5. Exon-4 Mutations Do Not Appear to Influence the Cellular Localization of KRAS Protein

**Figure 3:**
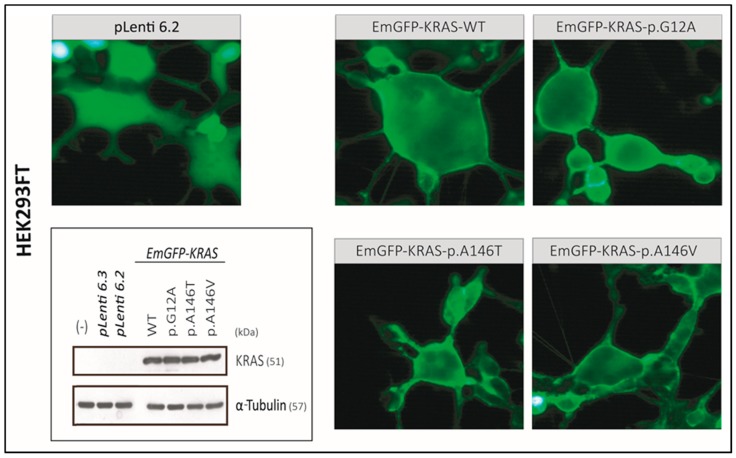
pLenti6.3 EmGFP-KRAS^WT/p.G12A/p.A146T/p.A146V^ expression in HEK293 cells as demonstrated by Western blot and fluorescence microscopy analysis. All KRAS-constructs are predominantly localized to the cell membrane of HEK293 cells compared to the pLenti6.2-EmGFP control 24 h after transfection. Fluorescence microscopy analysis was performed at 400× magnification. The corresponding original Western blots are shown in Figure S2. The raw intensities and the intensity ratios for each band are listed in Table S2.

To investigate whether the exon-4 mutations impact KRAS-binding to the membrane, which is reported to be essential for signal transduction [30], non-tumor HEK293 cells were stably transfected with pLenti6.3-EmGFP-KRAS^WT/p.G12A/p.A146T/p.A146V^ and the pLenti6.2-EmGFP vector control, respectively. Judged by fluorescence microscopy and by Western analysis, KRAS^WT^ and KRAS^p.G12A^, as well as both exon-4 mutants, were well expressed and—in contrast to the homogeneously distributed EmGFP signal—appeared to be preferentially localized to the cell membrane (Figure 3, Figure S2, Table S2).

### 2.6. Exon-4 Mutations Specifically Activate MEK and ERK in HEK293 Cells

**Figure 4:**
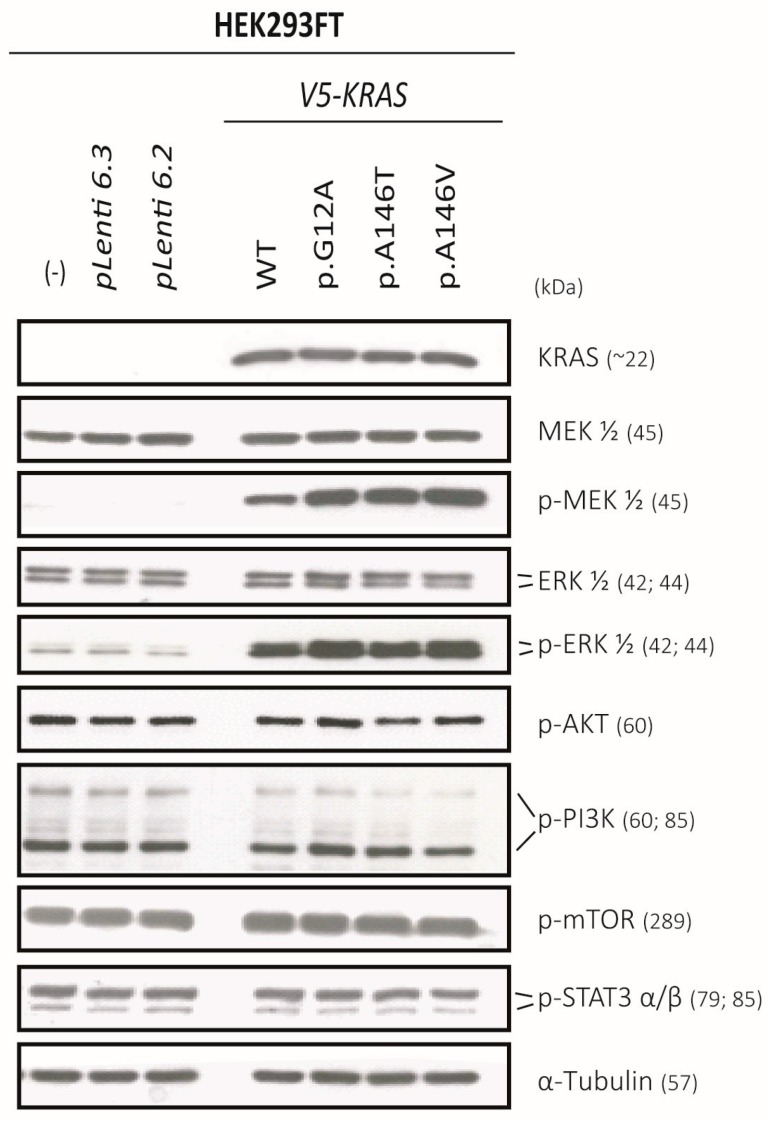
Representative expression and activation-levels of RTK effectors after transfection of KRAS^WT^ and KRAS^mut^ constructs into HEK293 cells. The corresponding original Western blots are shown in Figure S3. The raw intensities and the intensity ratios for each band are listed in Table S3.

The calculated molecular weight for pLenti6.3-V5-KRAS and pLenti6.3-EmGFP-KRAS proteins is ~25 kDa and ~51 kDa, respectively, which was confirmed by Western analysis (Figure 3 and Figure 4, Figure S3, Table S3). pLenti6.3-V5-KRAS-constructs were initially tested in HEK293 cells, in which they were well expressed (Figure 4, Figure S3, Table S3). While overexpression of KRAS^WT^ already had a significant activating effect on MEK, this activation was further increased for all KRAS-mutants tested (Figure 4, Figure S3, Table S3). The activation of ERK was, however, only slightly seen upon the overexpression of KRAS^G12A^ and KRAS^A146V^ (Figure 4, Figure S3, Table S3). No activation of PI3K, AKT, mTOR, or of STAT3 was observed upon the overexpression of KRAS^WT^ or KRAS^mut^ (Figure 4, Figure S3, Table S3).

### 2.7. The KRAS-Mutants KRAS^p.G12A/p.A146T/p.A146V^ Specifically Activate MEK/ERK-Signaling in the KRAS^WT^ MM Cell Lines JJN3 and OPM2 and Can Sustain AKT Signalling in OPM2

**Figure 5:**
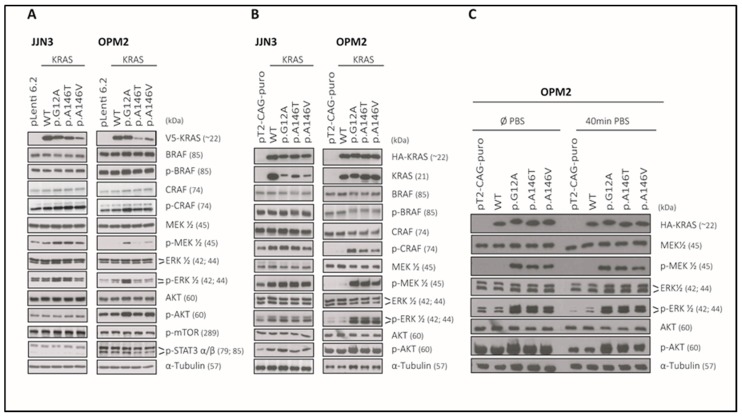
Representative expression and activation levels of RTK effectors upon the overexpression of KRAS^WT^ and mutant KRAS in MM cells, following lentiviral transfection of JJN3 and OPM2 cells (**A**). Stable transposition with Sleeping Beauty vectors of JJN3 and OPM2 cells, (**B**) or stable transposition with Sleeping Beauty vectors in OPM2 cells, which were either harvested from normal culture medium (Ø PBS) or after a 40 min washout period with PBS to curb FBS-mediated extracellular signals (40 min PBS), (**C**). Note: The same sample preparation was used for the blots shown for OPM2 in (**B**,**C**) (left part). The pictures for AKT and phospho-AKT staining are used in both figures. The corresponding original Western blots are shown in Figure S4A–C. The raw intensities and the intensity ratios for each band are listed in Table S4A–C. pLenti6.2: JJN3 or OPM2 cells transfected with the empty vector pLenti6.2. pT2-CAG-puro: JJN3 or OPM2 cells transfected with the empty vector pT2-CAG-puro.

Lentiviral transduction, as well as stable transposition with Sleeping Beauty vectors in the KRAS^WT^ cell lines JJN3 and OPM2 generated stable KRAS-overexpressing cells for all types of KRAS mutants (pLenti6.3-V5-KRAS^WT/p.G12A/p.A146T/p.A146V^; pSB-CAG-HA-KRAS^WT/p.G12A/p.A146T/p.A146V^) (Figure 5A–C, Figure S4A–C, Table S4A–C). Lentiviral transduction, however, did not lead to equal expression levels of all different KRAS-constructs in these cell lines (Figure 5A, Figure S4A, Table S4A). Therefore, we also tried the Sleeping Beauty-based approach using HA-tagged KRAS constructs in either CMV- or CAG-promotor-driven expression cassettes (only the latter experiments are shown in this paper due to insufficient expression results with the CMV-promotor constructs). These experiments led to similar results in JJN3 cells, with wild-type KRAS expressed at higher levels than the different point mutants. Sleeping Beauty-mediated transposition produced more equal expression results in OPM2 cells, however (Figure 5B,C, Figure S4B,C, Table S4B,C). Even though KRAS-mutants (specifically KRAS^p.A146T^ and KRAS^p.A146V^) were expressed at much lower levels compared to KRAS^WT^ following lentiviral transfection of JJN3 and OPM2 cells, anincrease or at least comparable levels of activated MEK and ERK (p-MEK and p-ERK) were observed upon the overexpression of KRAS^G12A/A146T/A146V^ compared to KRAS^WT^for both MM cell lines tested (Figure 5A, Figure S4A, Table S4A). The activation of MEK and ERK was particularly visible in Sleeping Beauty transposed OPM2 cells where KRAS^WT^ and all KRAS-mutants were equally expressed and corresponded well with other readouts for an activated RAS/MAPK cascade (phospho-ERK1/2; phospho-CRAF) in OPM2 (Figure 5B, Figure S4B, Table S4B). In JJN3, a slight activation of MEK but no activation of ERK was visible after transposition with Sleeping Beauty vectors. However, this might be due to the lower expression of KRAS^p.G12A/p.A146T/p.A146V^ compared to KRAS^WT^ that was also observed after lentiviral transfection. Notably, in addition to the strong intrinsic activation of the RAS/MAPK cascade, which was clearly observed in OPM2 cells after the transposition with Sleeping Beauty vectors, all KRAS mutants appeared capable to sustain high intrinsic levels of phospho-AKT, which became visible in the analyses of pathway activity after 40 min washout with phosphate buffered saline (PBS) and the resulting termination of fetal bovine serum (FBS) mediated pathway activation (Figure 5C, Figure S4C, Table S4C). Whereas this treatment led to a considerable attenuation of the high steady-state levels of phospho-AKT in either “normal” OPM2 cells expressing intrinsic levels of KRAS^WT^ or in OPM2 cells overexpressing KRAS^WT^, it barely affected the signal in mutant KRAS transfected cells (Figure 5C, Figure S4C, Table S4C). Notably, no activation of mTOR, STAT3, or BRAF was observed upon the overexpression of KRAS^WT^ or any KRAS-mutant in JJN3 and OPM2 cells (Figure 5A,B, Figure S4A,B, Table S4A,B). In summary, although consistent overexpression of KRAS^WT^ and KRAS^p.G12A/p.A146T/p.A146V^ proteins could only partially be achieved, our approach using two different stable transfection systems in two KRAS-wildtype MM cell line models still proved the capacity for activation of two major oncogenic pathways not only for the classical p.G12A mutant, but also for both p.A146 mutants.

## 3. Discussion

Mutations in *KRAS* and *NRAS* are the most frequently detected point mutations in MM and it was shown that survival of MM cell lines depends on oncogenic RAS [4,9,10]. After more than 30 years of research aimed at developing useful RAS inhibitors, AMG 510, an inhibitor specifically for p.G12C mutant KRAS, is now being tested in a clinical trial and is the first hope for targetting oncogenic KRAS therapeutically [1]. However, only a few studies exist in MM that investigated the clinical role of *KRAS*-mutations after the introduction of novel agents such as bortezomib and lenalidomide, which nowadays are routinely included in MM therapies [31], and all of these studies focused on the role of *KRAS*-mutations in relapsed/refractory disease [4,18]. Moreover, functional investigations on oncogenic KRAS in MM are rather limited. Specifically, no expression studies have so far been reported that have investigated the role of the common exon-2 KRAS mutation p.G12A and the rare exon-4 KRAS-mutations p.A146T and p.A146V regarding their impact on different survival pathways. To our knowledge, only shRNA-mediated KRAS-knockdown experiments in MM cell lines [10] and overexpression experiments of the exon-2 mutant p.G12V in the MM cell line ANBL6 have been performed, but the latter analysis did not describe the influence on classical survival pathways such as the MEK/ERK- and the PI3K/AKT-pathways [32,33]. Thus, our study aims to correlate the occurrence of clonal and subclonal *KRAS*-mutations in a MM cohort at diagnosis, treated with bortezomib-containing induction regimens, with common clinical and cytogenetic parameters, and to study the impact of the KRAS^p.G12A^, KRAS^p.A146T^, and KRAS^p.A146V^ mutations on survival pathways in recombinant HEK293 and MM cell line models.

Our sequencing approach in the study cohort of the DSMM XI trial confirmed the reported distribution of clonal and subclonal *KRAS*-mutations [9,19,20,22,34] and the results obtained by correlation of the *KRAS*-mutation profile with survival are in line with previous observations in bortezomib-treated patients at relapse [4,18]. *KRAS*-mutations did not have an influence on survival in bortezomib-treated patients at diagnosis. However, in contrast to the findings by an in silico analysis of the CoMMpass dataset [14], this was still evident after the separation into clonal and subclonal prevalence in our current dataset. Consistent with this finding, no association was observed with high-risk (e.g., del17p), intermediate-risk (e.g., t(4;14)), or standard-risk (e.g., t(11;14)) cytogenetic parameters. This is in line with recent reports [13,18], but does not support the findings of Rasmussen et al., who observed a positive-correlation of *KRAS*-mutations with high CCND1 expression (e.g., t(11;14)) and a negative correlation with the occurrence of the translocation t(4;14) [17]. Moreover, in our cohort we found no evidence for co-occurrence of *KRAS*- and *DIS3*-mutations as previously described, which might be due to the limited number of patients studied in the current analysis [20].

In summary, our analysis of the prognostic role of KRAS-mutations in a study cohort with newly diagnosed MM, uniformly treated with three cycles of VCD and subsequent stem cell mobilization, high-dose chemotherapy, and autologous stem cell transplantation, did not reveal a significant correlation with survival.

Interestingly, however, the exon-4 *KRAS* mutation p.A146T detected in AMO1 was accompanied by a CN-gain (CN-status = 4) and a 2.3-fold higher mRNA-expression of *KRAS* reminiscent of the situation in colorectal cancer cell lines [24]. An shRNA-mediated KRAS-knockdown in this cell line, however, had no influence on MEK-/ERK-signaling while a KRAS knockdown in the KRAS^p.G12A^ mutant MM cell line MM1.S, which is neither affected by *KRAS* CN-gains nor increased mRNA expression, led to reduced ERK-activity and survival [10], coherent with the activation of the RAS/MAPK pathway in our current overexpression experiments of KRAS^p.G12A^ in HEK293, JJN3 and OPM2 cells. One reason for this inconsistency among the two cell lines might be that the amount of shRNA used was sufficient for a KRAS CN-status of 2 and moderate KRAS mRNA-expression, but not sufficient for a KRAS CN-stauts of 4 and increased KRAS mRNA-expression. Activation of ERK by KRAS^p.G12A^ was, however, not seen by Xu and colleagues in primary MM using an immunohistochemistry-based approach [35]. It remains unclear whether this apparent lack of activation results from a different technical approach, whether the expression of KRAS^p.G12A^ has a different effect in primary MM, or whether the magnitude of its effects is dependent on the context within the individual pattern of oncogenic lesions present in the affected cells. Interestingly, the common KRAS^p.G12A^ as well as the rare exon-4 mutations KRAS^p.A146T^ and KRAS^p.A146V^ specifically activated MEK/ERK- and AKT-signaling compared to KRAS^WT^, although the activation of ERK and AKT in the lentiviral approach was not as clear as in the Sleeping Beauty experiments, which might be due to the relatively low expression levels of the pLenti6.3-KRAS^p.A146T/p.A146V^ constructs. Furthermore, ERK- and MEK-activation were less prominent in JJN3 overexpressing the KRAS-mutants and no activation of AKT was observed in this MM cell line. Partly, this uneven pattern may be related to the strong differences in expression levels encountered for the different KRAS constructs, and partly this may be a consequence of the different patterns of oncogenic lesions affecting the RAS/MAPK and AKT-pathways in these cell lines [26,36]. For example, JJN3 cells appear to exist in different versions, and those used in this experiment harbour an activating NRAS mutation [10] potentially impinging on the potential for the ectopic constructs to leave their mark. OPM2, on the other hand, is characterized by the presence of an activating K650E mutation in FGFR3 and by deficiency in *PTEN*. Especially the latter lesion, which affects the rate at which AKT can be dephosphorylated, is responsible for the high constitutive levels of phosphorylated AKT in this cell line [37] and it may be the decreased rate of AKT dephosphorylation that permits the detection of the intrinsic activation of this pathway through the introduced RAS-mutants under conditions of serum deprivation in OPM2 cells. However, while we could clearly demonstrate that KRAS^p.G12A^, KRAS^p.A146T^, and KRAS^p.A146V^ activate MEK-/ERK-signaling and AKT in OPM2 cells, we did not observe an influence of oncogenic KRAS on mTOR-activation in either OPM2 or JJN3. CRAF, on the other hand, appears to play a role in relaying oncogenic KRAS activity, since the expression of oncogenic KRAS in MM cells clearly affected the levels of activated CRAF. These observations are mechanistically consistent with previous findings that described a switch from BRAF to CRAF signaling upon the expression of mutant KRAS in melanoma cells [38,39]. Our functional analyses of a common exon-2 (p.G12A) and of the uncommon exon-4 (p.A146T, p.A146V) KRAS mutations therefore underscored their context-dependent capacity to activate and/or sustain oncogenic RAS/MAPK and AKT signaling in MM cells. Unfortunately, normal plasma cells are not an accessible model to test the functional consequences of the uncommon KRAS exon-4 mutations, nor is it clear which MM cell lines would provide definitive answers to assess their transformative potential, since all display lesions that already affect RAS-dependent pathways. Additionally, our difficulties to maintain constant and similar expression levels of KRAS-constructs over extended periods of time in MM cells precluded clonal competition analyses which might otherwise have provided some clues as to the potential gain of fitness associated with such mutations. However, when characterized in MM patients (our data, the CoMMpass dataset), and in the one case known from an MGUS sample [40] KRAS exon-4 mutations were always present with high allele frequency at DNA and RNA level (e.g., higher than for the common exon-2 and exon-3 mutations p.G12 and p.Q61), suggesting that they are a major determinant in the rise to clonal dominance. There is, however, also an interesting MM case described by Corre et al. [34] in which a p.A146 KRAS mutation detected with a realtively high VAF of 25% at diagnosis became superseded by an initially minor KRAS p.G13D clone at relapse.

In conclusion, these analyses suggest that p.A146 mutations merit full consideration when assessing patients’ tumor genetics and could also be considered for the development of further specific KRAS-inhibitors.

## 4. Materials and Methods

### 4.1. Patient Specimens and Human Cell Lines

The 80 MM patients of the DSMM XI trial, included in this study, were treated with VCD, and subsequent stem cell mobilization, high-dose chemotherapy, and autologous stem cell transplantation [29,41,42]. Primary MM cells were isolated from bone marrow aspirates by the CD138+ microbead procedure (Miltenyi Biotec, Bergisch Gladbach, Germany) [43]. Peripheral blood mononuclear cells of the same patients served as corresponding normal controls [29]. Approval for the trial and the accompanying research projects was obtained from the local ethics commitee (ref. no. 18/09, approval renewed: 09 March, 2009, reference number AZ 76/13, date of approval: 18.04.2013), and by the University of Ulm (application number: 307/08, date of approval: 21 January 2009).

The human MM cell lines L363, JJN3, OPM2, U266, AMO1, KMS12BM, MOLP8, NCIH929, and RPMI8226 were purchased from the “Deutsche Sammlung von Mikroorganismen und Zellkulturen GmbH” (DSMZ, Braunschweig, Germany), MM1.S from LGC Biolabs (Wesel, Germany), and INA6 was a kind gift from Professor Martin Gramatzki (University Hospital Schleswig-Holstein, Kiel, Germany). The human embryonic kidney cell line HEK293FT was obtained from Thermo Fisher Scientific (Darmstadt, Germany). MM cells were cultured as described previously [29] and HEK293FT-cells were grown in DMEM supplemented with 0.5% fetal bovine serum (FBS) and 6 mM L-glutamine. To ensure long-term cell line authenticity, newly purchased cells were immediately expanded and up to 40 aliquots were cryo-conserved (“stock bank”). From one of these aliquots, a second bank of up to 30 aliquots (“working bank”) was then established. Subsequently, cell cultures were always retired after 3–4 months and freshly re-instated from working bank aliquots as needed (“dead-end cell culture”). Diminished working banks were replenished by repeating the procedure with the next stock bank aliquot. Cultures used for preparation of the banks, as well as cultures in use for more than a month, were monitored for mycoplasma negativity using the VenorGEM One-Step kit (Minerva Biolabs, Berlin, Germany).

### 4.2. Amplicon Generation and Sequencing

A library of the whole coding sequence of *KRAS* (NM_004985.4, NM_033360.3) was prepared using 50 ng DNA per sample. Amplicons for the coding regions of *KRAS* were generated using exon-specific primers (Table S5) for all MM samples and MM cell lines with the 48-48 Access ArrayTM IFC using the Fluidigm FCI Cycler System (Fluidigm, Amsterdam, The Netherlands) and sequenced in a 12-plex format with the Roche 454 GS Junior (Roche, Mannheim, Germany), as described previously [29]. Sequencing data are deposited at the European Genome-phenome Archive (EGA; http://www.ebi.ac.uk/ega/), which is hosted at the EBI, under accession number EGAS00001003945.

### 4.3. Sequencing Data Analysis and Technical Verification

Sequencing data were analyzed with the GS Run Processor and the GS Amplicon Variant Analysis software (Roche) and SNVs were annotated, filtered, and assigned to major and minor subclones as described previously [29].

Moreover, the frequency of SNVs occurring in KRAS within the CoMMpass dataset was determined, which was generated as part of the Multiple Myeloma Research Foundation Personalized Medicine Initiatives (Initiatives 2014) [25].

### 4.4. Sanger Sequencing and High Resolution Meling (HRM)

All SNVs that were present in at least 5% of reads were technically verified by Sanger sequencing (VAF of >20%) and HRM (VAF <20%) using the LightCycler 480 High Resolution Melt Master Kit (Roche) as described previously [29,41]. Primers used for these purposes are listed in Table S6.

### 4.5. Statistical Analysis

Statistical analysis was performed using GraphPad Prism 8 software. Correlations of *KRAS*-mutations with cytogenetic parameters and the clinical parameters gender, light chain, and event were done with cross-tabulations in combination with Fisher’s exact test for significance. Correlation with the clinical parameters heavy chain, stage, and response to treatment were done with the two-way-ANOVA and age at diagnosis using the Welch test. Survival statistics were calculated using Kaplan-Meier curves with log-rank tests for significance.

### 4.6. Generation of Transient and Stable KRAS-WT and KRAS-Mutant (p.G12A, p.A146T, p.A146V) Overexpression and of KRAS-Knockdown Cell Lines

**Figure 6:**
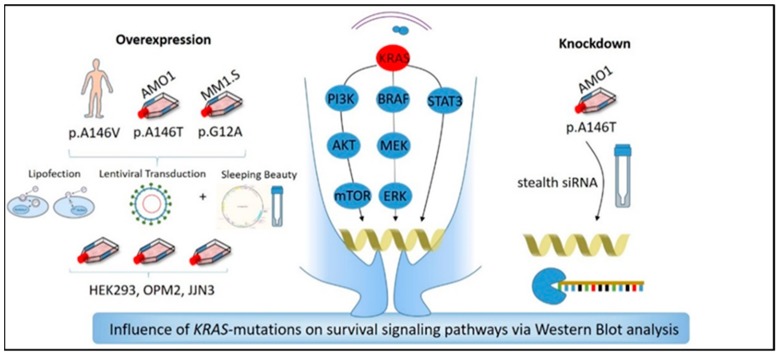
Experimental approaches to investigate the functional role of the KRAS exon-4 mutations p.A146T and p.A146V in comparison to the exon-2 mutation p.G12A.

Functional analyses of KRAS exon-2 and exon-4 mutations required stable and transient KRAS-overexpression and transient KRAS-knockdown (Figure 6). Stable overexpression in MM cells was achieved by lentiviral transduction or transfection (electroporation)/transposition with Sleeping Beauty vectors. Transient overexpression in HEK293FT cells was achieved using lipofection, and transient knockdown of KRAS was obtained by an siRNA-mediated approach.

### 4.7. Isolation of RNA and cDNA Synthesis

RNA was isolated using the Qiagen All Prep Kit and transcribed into cDNA using an oligo (dT)_18_ primer and the First Strand cDNA Synthesis Kit (Thermo Fisher Scientific).

### 4.8. SNP 6.0 and HGU133 Plus 2.0 Microarray Analysis

CN-profiles and gene expression profiles of the cell lines AMO1, U266, L363, OPM2, JJN3, and MM1.S were generated using SNP 6.0 and HG U133 plus 2.0 arrays (Thermo Fisher Scientific), respectively. The hybridization of RNA and DNA to these arrays as well as washing and scanning was performed according to the manufacturer’s instructions. Gene expression and CN-data were processed with the Affymetrix Expression Console and the Affymetrix Genotyping Console Software (Thermo Fisher Scientific), as described previously [44,45].

### 4.9. Generation of Donor and Expression Vectors for Lentiviral Transduction

The stable KRAS-WT and KRAS-mutant overexpression MM cell lines OPM2 and JJN3 were generated with the ViraPower™ Lentiviral Expression System (Thermo Fisher Scientific, Darmstadt, Germany). All steps were performed according to the guidelines of the manufacturer, if not otherwise specified. Expression cassettes for the fusion-proteins attB-V5-KRAS and EmGFP-KRAS were generated in a two-step fusion-PCR using different primer combinations (Table S7) and the FastStart High Fidelity PCR System (Roche, Mannheim, Germany) as depicted in Figure S5. In a first PCR step, primers a and b were used to amplify V5-*KRAS* with attb overhang, primers g and b to amplify *KRAS* with EmGFP and atbb overhang, as well as primers e and h to amplify EmGFP with *KRAS* overhang (Table S7). A second PCR step was then performed to add the complete attb-sites to the V5-*KRAS* and the pre-final EmGFP *KRAS* construct and to fuse EmGFP to *KRAS* to generate the final attb-EmGFP-*KRAS* construct. The Vivid Colors pLenti6.2-GW/EmGFP-vector served as a template for the EmGFP-construct, and the *KRAS*-cDNAs of the MM-patient P30 and the cell lines AMO1, MM1.S as templates for the *KRAS*-mutants p.A146V, p.A146T, and p.G12A, respectively. After technical verification of the amplified products using a 1% agarose gel, they were purified with the PEG purification protocol from Thermo Fisher Scientific (Darmstadt, Germany) and Sanger-sequenced, to confirm the presence of the respective *KRAS*-mutations. For the generation of the entry vectors, pDONOR221-V5-*KRAS* and pDONOR221-EmGFP-*KRAS* 50 fmol of both the attB- PCR-product and the pDONOR221 were used for the BP-recombination. Subsequently, 1 µL of this reaction was transformed into TOPO10 *E. coli* cells. Clones were picked and verified by Sanger sequencing using the M13 forward primer that binds within the pDONOR221 and the reverse primer b that binds to the *KRAS*-cDNA (Figure S5, Table S7), as well as by restriction digestion using the enzymes EcoRI and PvuI which cut the vector pDONOR221 and KRAS-cDNA, respectively. The successfully cloned pDONOR221-V5-*KRAS* and pDONOR-EmGFP-*KRAS* constructs were then used in the LR-recombination together with the expression vector pLenti6.3 to generate the recombinant expression vectors pLenti6.3-V5-*KRAS* and pLenti6.3-EmGFP-*KRAS*. Notably, the non-mutated WT-allele of AMO1 was used as *KRAS*-WT in this reaction. Then, 2 µL of the LR-reaction were subsequently transformed into OneShotStbl3 *E. coli* cells and positive clones identified via restriction digestion using the enzymes EcoRI-HF which cuts sites within both *KRAS* and pLenti6.3 and Mfel-HF which cuts at a site only present in pLenti6.3. Clones carrying plasmids with the correct restriction pattern were grown overnight (ON) and verified by Sanger sequencing using the CMV forward primer and the reverse primer b (Figure S5, Table S7) which bind to pLenti6.3 and *KRAS*, respectively.

### 4.10. Virus Production in HEK293FT Cells

One day prior to transfection, 6 × 10^5^ HEK293FT cells were plated per well in 6-well plates. Following incubation ON, the medium was replaced by 500 µL Opti-MEM I containing 10% FBS. 3.6 µL of Lipofectamine 2000 (Thermo Fisher Scientific, Darmstadt, Germany), which were were then diluted with 150 µL Opti-MEM I, vortexed, and incubated for 5 min at room temperature (RT). A mixture containing 0.3 µg expression vector, 0.9 µg of the *lentiviral packaging mix* (ViraPower™ Lentiviral Expression System, Thermo Fisher Scientific, Darmstadt, Germany), and 150 µL Opti-MEM I+Lipofectamine 2000 was then incubated for another 20 min at RT and added to the HEK293FT cells followed by incubation ON. The experiments for each expression construct were set up in triplicates. pLenti6.2 was used as a positive control. After 24 h the medium was replaced by 1 mL DMEM and incubation continued for another 24 h. The supernatant was removed, pooled for each construct, and centrifuged for 5 min at 1500 rpm.

### 4.11. Viral Transduction of the MM Cell Lines OPM2 and JJN3

1 × 10^6^ cells/well in 3 mL medium were plated in wells of a 6-well plate and 1 mL of the virus-suspension was added to the cells which resulted in a multiplicity of infection (MOI) of ≤1. Following overnight incubation, cells were transduced once more with 1 mL of the virus-suspension and again incubated for 24 h. Duplicates were pooled and cells with EmGFP-constructs were FACS-sorted and selected for low expression levels. Cells with V5-constructs were pooled and treated with 3 µg/mL blasticidin for 14 days. The medium containing blasticidin was replaced every three days.

### 4.12. Generation of Stable Overexpression Cell Lines Using the Sleeping Beauty System

The coding sequence for WT and mutant KRAS was PCR-amplified off the above-described lentiviral vectors with primers introducing NheI and NotI sites at the 5-prime and 3-prime ends, respectively, and cloned into a modified pBluescript II vector adding a coding sequence for an aminoterminal HA-tag. After sequence verification, the constructs were excised by digestion with SpeI and NotI and subcloned into NheI/NotI acceptor sites within a CAG-promotor-driven expression cassette of a modified Sleeping Beauty vector (pT2-CAG-puro). Subsequently, the recombinant plasmids containing KRAS-WT or the KRAS-mutants p.G12A, p.A146T, and p.A146V, respectively, were transfected together with a transposase expression plasmid into OPM2 and JJN3 cells via electroporation according to previously published protocols [46]. Electroporated cells were cultured under standard conditions with puromycin (2 µg/mL for 10 days) until upgrowth of stably transfected polyclonal cultures, and aliquots were cryoconserved for longer-term storage.

### 4.13. Transfection of HEK293FT Cells with Lipofectamine 2000

6 × 10^5^ HEK293FT cells/well were plated in wells of a 6-well plate. Following incubation ON the medium was replaced by 500 µL Opti-MEM I. A mixture containing 150 µL Opti-MEM I and 12 µL Lipofectamine 2000 was incubated for 5 min at RT and then supplemented with another suspension containing 150 µL Opti-MEM I and 2.5 µg of the respective pLenti6.3-*KRAS*-construct or an empty-vector control. Following 20 min incubation at RT, the mixture was added to the cells which were subsequently incubated ON at 37 °C. Proteins were extracted from whole cell lysates to study the influence of WT- and mutant-*KRAS* overexpression on different signaling pathways. The expected molecular weight of the fusion-proteins was determined in advance using Expasy (https://www.expasy.org/) and Sciencegateway (https://www.sciencegateway.org/).

### 4.14. FACS Analysis

Transduction efficiency, as well as numbers of dead and living cells, were determined by FACS analysis using a BD FACS Canto II (BD Bioscience, Heidelberg, Germany) on cells transduced with EmGFP-expression virus or transfected with Alexa 488 labeled siRNA or by incubation in 300 µL FACS-buffer containing 1 µL propidiumiodide (PI) and 1 µL annexin V-APC stock solutions.

### 4.15. SDS PAGE and Immunoblotting

For the preparation of whole cell lysates from the *KRAS*-WT and mutant transfected/transduced cell lines, 250 µL lysis buffer were added to 1 × 10^7^ cells. A 1:10 pre-dilution of the supernatant in H_2_O was then taken to determine the protein concentration by Bradford assay at a wavelength of 595 nm with a FLUOstar Omega (BMG Labtech, Ortenberg, Germany). Then, 30 µg of protein was diluted with 4x loading dye and heated to 95 °C for 5 min for sodium dodecylsulfate polyacrylamide gel electrophoresis (SDS-PAGE) using 6% stacking and 10% separation gels. To determine the molecular weight, the prestained (#26619, 10–250 kDa) or unstained (#26614, 10–200 kDa) protein ladders from ThermoFisher Scientific were loaded on a separate lane of each polyacrylamide gel. After protein transfer to nitrocellulose membranes using the wet western transfer system from Peqlab Biotechnologies (Erlangen, Germany), the membranes were blocked with either 5% milk powder in Tris-buffered saline with 0.1% Tween (TBS-T) or 5% BSA in TBS-T for 1 h at RT. After blocking of the membrane, the respective primary antibody (KRAS, MEK, p-MEK1/2, ERK1/2, p-ERK1/2, p-STAT3, AKT, p-AKT, BRAF, p-BRAF, CRAF, p-CRAF, p-mTOR, p-PI3K, tubulin α, anti-V5) was added and incubated at 4 °C ON (Table S8). After incubation, membranes were washed 3x 10 min with TBS-T before the corresponding secondary antibody (rabbit anti-mouse (#P0260) diluted 1:10000 in TBS-T; anti-mouse (#7076) diluted 1:3000 in 5%- BSA/TBS-T; anti-rabbit (#7074), diluted 1:2000 in 5% BSA/TBS-T) was added for 1–2 h at RT (Table S8). Following the incubation with secondary antibody, membranes were washed 3x 10 min with TBS-T. SuperSignal West Pico Chemiluminescent Substrate (Thermo Fisher Scientific, Darmstadt, Germany) was added to the membrane for 1 min followed by the visualization of protein signals using X-ray film. Intensity ratios were determined using the ImageJ software.

## 5. Conclusions

Our current analysis of the prognostic role of KRAS-mutations in a NDMM patient cohort, treated uniformely with novel agents, did not reveal a significant correlation with survival. Functional analysis of the common p.G12A exon-2 mutation and the rare exon-4 mutations p.A146T and p.A146V, however, underscored their capacity to activate and/or sustain oncogenic Ras/MAPK and AKT signaling in MM cells, confirming the importance of a mutation-driven activation of MEK-/ERK-signaling in this disease [47]. These results indicate that the development of further mutation-specific KRAS-inhibitors, such as the p.G12C-inhibitor AMG 510, could be of great value for the individual treatment of KRAS-mutant MM patients.

## Figures and Tables

**Table 1:** Correlation of the *KRAS* mutation-status with classic cytogenetic parameters. mut: mutation, WT: wild type.

Cytogenetic Parameters	*KRAS* Mut, *n* = 16	*KRAS* WT, *n* = 64	*p*-Value
13q deletion; no, yes	9, 7	29, 35	0.577
17p deletion; no, yes	14, 2	50, 14	0.504
1q gain; no, yes	12, 4	41, 22	0.559
9q gain; no, yes	10, 6	33, 31	0.577
t(4;14); no, yes	12, 4	48, 16	1
t(11;14); no, yes	12, 4	49, 15	1
t(14;16); no, yes	15, 1	61, 2	0.498
t(8;14); no, yes	14, 2	61, 1	0.105
t(14;20); no, yes	16, 0	62, 0	1
*DIS3* mut; no, yes	15, 1	56, 8	0.679

## Supplementary Materials

The following are available online at https://www.mdpi.com/2072-6694/12/2/455/s1, Figure S1: Correlation of major and minor *KRAS* subclone mutations with cytogenetic parameters, response to therapy and survival, Figure S2: Original Western Blots corresponding to Figure 3, showing pLenti6.3 EmGFP-KRAS^WT/p.G12A/p.A146T/p.A146V^ expression in HEK293 cells as demonstrated by Western blot analysis. Negative (−): JJN3 or OPM2 cells without vector. Negative (+): JJN3 or OPM2 cells without vector, but treated with transfection reagents. pLenti6.3: JJN3 or OPM2 cells transfected with empty vector pLenti6.3. pLenti6.2: JJN3 or OPM2 cells transfected with the empty vector pLenti6.2, Figure S3: Original Western Blots corresponding to Figure 4 showing the expression and activation-levels of RTK effectors after transfection of KRAS^WT^ and KRAS^mut^ constructs into HEK293 cells. Negative (−): HEK293 cells without vector. Negative (+): HEK293 cells without vector, but treated with transfection reagents. pLenti6.3: JJN3 or OPM2 cells transfected with empty vector pLenti6.3. pLenti6.2: JJN3 or OPM2 cells transfected with the empty vector pLenti6.2, Figure S4A: Original Western Blots corresponding to Figure 5A, showing the expression and activation levels of RTK effectors upon the overexpression of KRAS-WT and mutant KRAS in MM cells, following lentiviral transfection in JJN3 and OPM2 cells. Negative (−): JJN3 or OPM2 cells without vector. pLenti6.2: JJN3 or OPM2 cells transfected with the empty vector pLenti6.2, Figure S4B: Original Western Blots corresponding to Figure 5B, showing the expression and activation levels of RTK effectors upon the overexpression of KRAS-WT and mutant KRAS in MM cells, following stable transposition with Sleeping Beatuy vectors in JJN3 and OPM2 cells (B). Negative: JJN3 or OPM2 cells without the vector pT2-CAG-puro. pT2-CAG-puro: JJN3 or OPM2 cells transfected with the empty vector pT2-CAG-puro, Figure S4C: Original Western Blots corresponding to Figure 5A–C, showing the expression and activation levels of RTK effectors upon the overexpression of KRAS-WT and mutant KRAS in MM cells, following stable transposition with Sleeping Beatuy vectors in OPM2 cells, which were either cultured in full cell culture medium as described in materialand methods (Ø PBS) or kept 40 min in PBS without the addition of FBS (40 min PBS). Negative: JJN3 or OPM2 cells without the vector pT2-CAG-puro. pT2-CAG-puro: JJN3 or OPM2 cells transfected with the empty vector pT2-CAG-puro, Figure S5: Strategy of the two-step fusion-PCR using different primer combinations to generate the fusion-proteins attB-V5-KRAS and EmGFP-KRAS (Table S III), Table S1: Clinical parameters of MM patients with and without KRAS mutations, Table S2: Raw intensities and intensity ratios of Western blot bands as well as fold change expression/activation, corresponding to Figure 3 and Figure S2, Table S3: Raw intensities and intensity ratios of western blot bands as well as fold change expression/activation of proteins, corresponding to Figure 4 and Figure S3, Table S4: Raw intensities and intensity ratios of western blot bands as well as fold change expression/activation of proteins, corresponding to Figure 5A–C and Figure S4A–C, Table S5: Exon specific KRAS-primers to generate the amplicons with the 48-48 Access ArrayTM IFC using the Fluidigm FCI Cycler System (Fluidigm, Amsterdam, The Netherlands), Table S6: Primers for validation with High Resolution Melting, Table S7: Primers for fusion PCR and for the validation of subsequent cloning procedures, Table S8: Antibodies used for Western blotting and FACS analysis.
