# Supplementary material for: Exon-4 Mutations in KRAS Affect MEK/ERK and PI3K/AKT Signaling in Human Multiple Myeloma Cell Lines
Source: Cancers (Basel). 2020 Feb 16;12(2):455. doi: 10.3390/cancers12020455 (PMC7072554; doi:10.3390/cancers12020455)
Supplement: Supplementary file 1 [file cancers-12-00455-s001.zip › Supplementary material/Figure S2_original western blots for Figure 3_revised.pptx]

## Slide 1
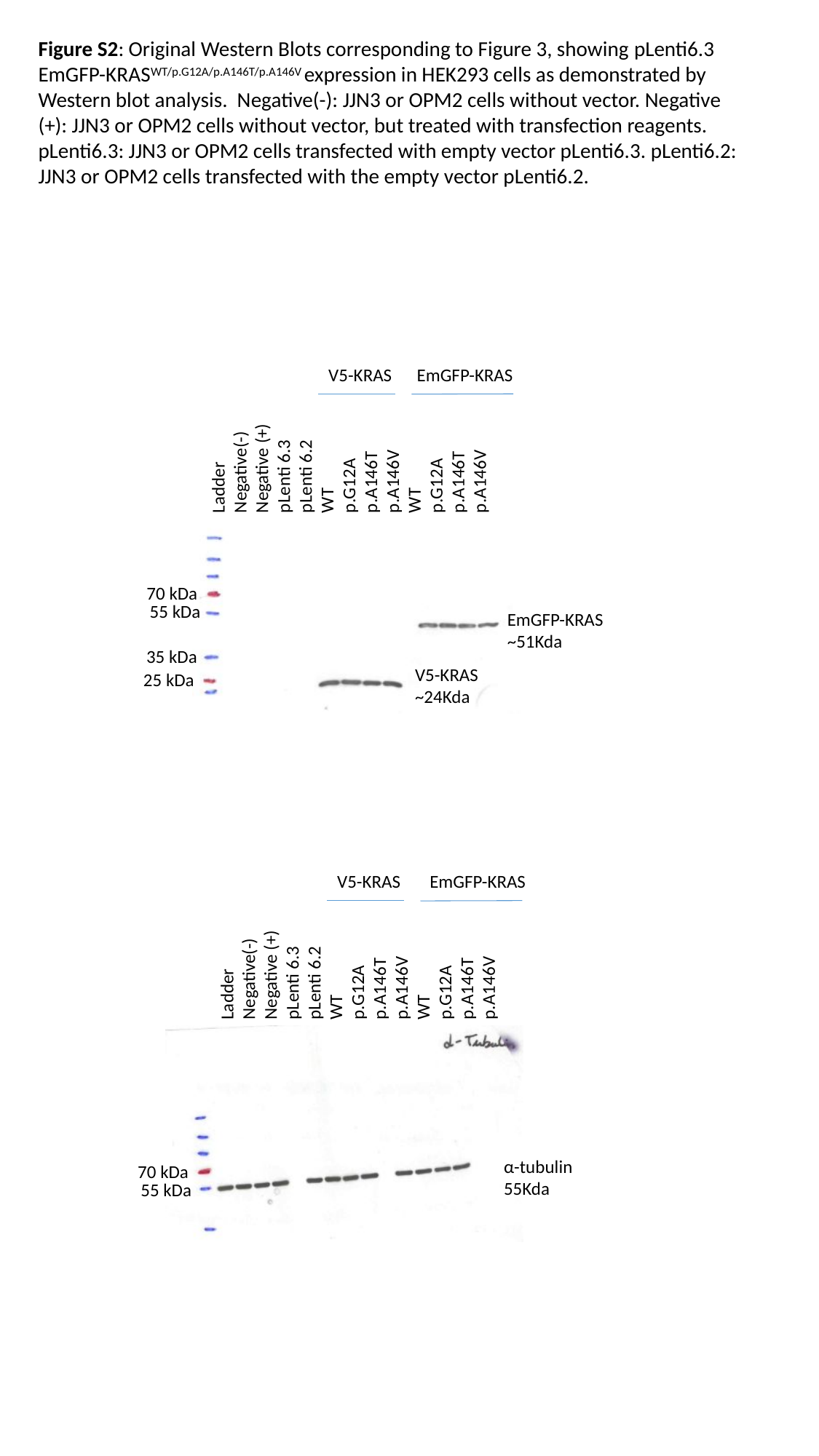

Figure S2: Original Western Blots corresponding to Figure 3, showing pLenti6.3 EmGFP-KRASWT/p.G12A/p.A146T/p.A146V expression in HEK293 cells as demonstrated by Western blot analysis. Negative(-): JJN3 or OPM2 cells without vector. Negative (+): JJN3 or OPM2 cells without vector, but treated with transfection reagents. pLenti6.3: JJN3 or OPM2 cells transfected with empty vector pLenti6.3. pLenti6.2: JJN3 or OPM2 cells transfected with the empty vector pLenti6.2.
Ladder
Negative(-)
Negative (+)
pLenti 6.3
pLenti 6.2
WT
p.G12A
p.A146T
p.A146V
WT
p.G12A
p.A146T
p.A146V
V5-KRAS EmGFP-KRAS
70 kDa
55 kDa
EmGFP-KRAS
~51Kda
35 kDa
V5-KRAS
~24Kda
25 kDa
Ladder
Negative(-)
Negative (+)
pLenti 6.3
pLenti 6.2
WT
p.G12A
p.A146T
p.A146V
WT
p.G12A
p.A146T
p.A146V
V5-KRAS EmGFP-KRAS
α-tubulin
55Kda
70 kDa
55 kDa
