# Supplementary material for: Exon-4 Mutations in KRAS Affect MEK/ERK and PI3K/AKT Signaling in Human Multiple Myeloma Cell Lines
Source: Cancers (Basel). 2020 Feb 16;12(2):455. doi: 10.3390/cancers12020455 (PMC7072554; doi:10.3390/cancers12020455)
Supplement: Supplementary file 1 [file cancers-12-00455-s001.zip › Supplementary material/Figure S3_original western blots for Figure 4_revised.pptx]

## Slide 1
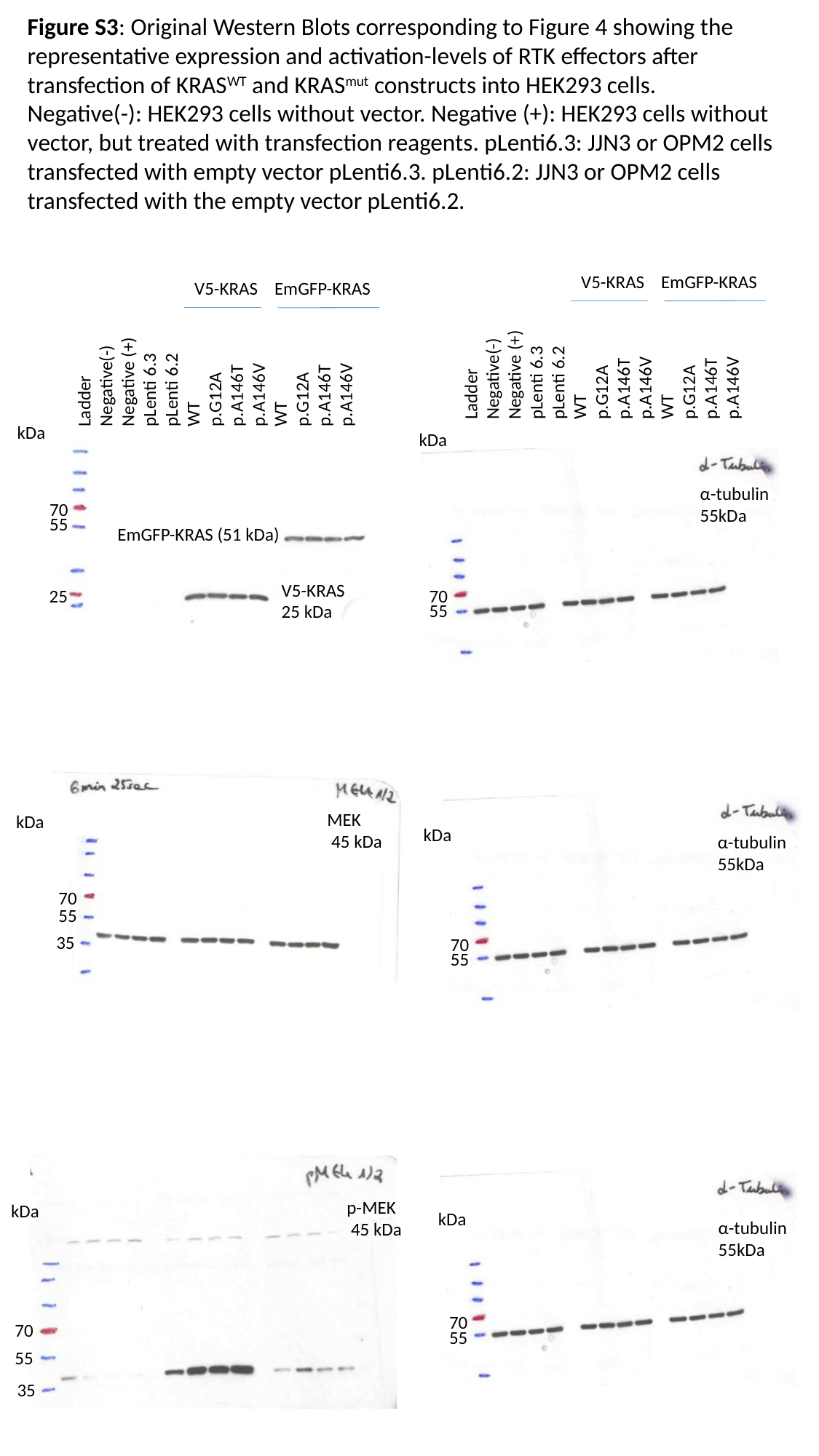

Figure S3: Original Western Blots corresponding to Figure 4 showing the
representative expression and activation-levels of RTK effectors after
transfection of KRASWT and KRASmut constructs into HEK293 cells.
Negative(-): HEK293 cells without vector. Negative (+): HEK293 cells without
vector, but treated with transfection reagents. pLenti6.3: JJN3 or OPM2 cells
transfected with empty vector pLenti6.3. pLenti6.2: JJN3 or OPM2 cells
transfected with the empty vector pLenti6.2.
Ladder
Negative(-)
Negative (+)
pLenti 6.3
pLenti 6.2
WT
p.G12A
p.A146T
p.A146V
WT
p.G12A
p.A146T
p.A146V
Ladder
Negative(-)
Negative (+)
pLenti 6.3
pLenti 6.2
WT
p.G12A
p.A146T
p.A146V
WT
p.G12A
p.A146T
p.A146V
V5-KRAS EmGFP-KRAS
V5-KRAS EmGFP-KRAS
kDa
kDa
α-tubulin
55kDa
70
55
EmGFP-KRAS (51 kDa)
V5-KRAS
25 kDa
25
70
55
MEK
 45 kDa
α-tubulin
55kDa
kDa
kDa
70
55
35
70
55
p-MEK
 45 kDa
α-tubulin
55kDa
kDa
kDa
70
70
55
55
35

## Slide 2
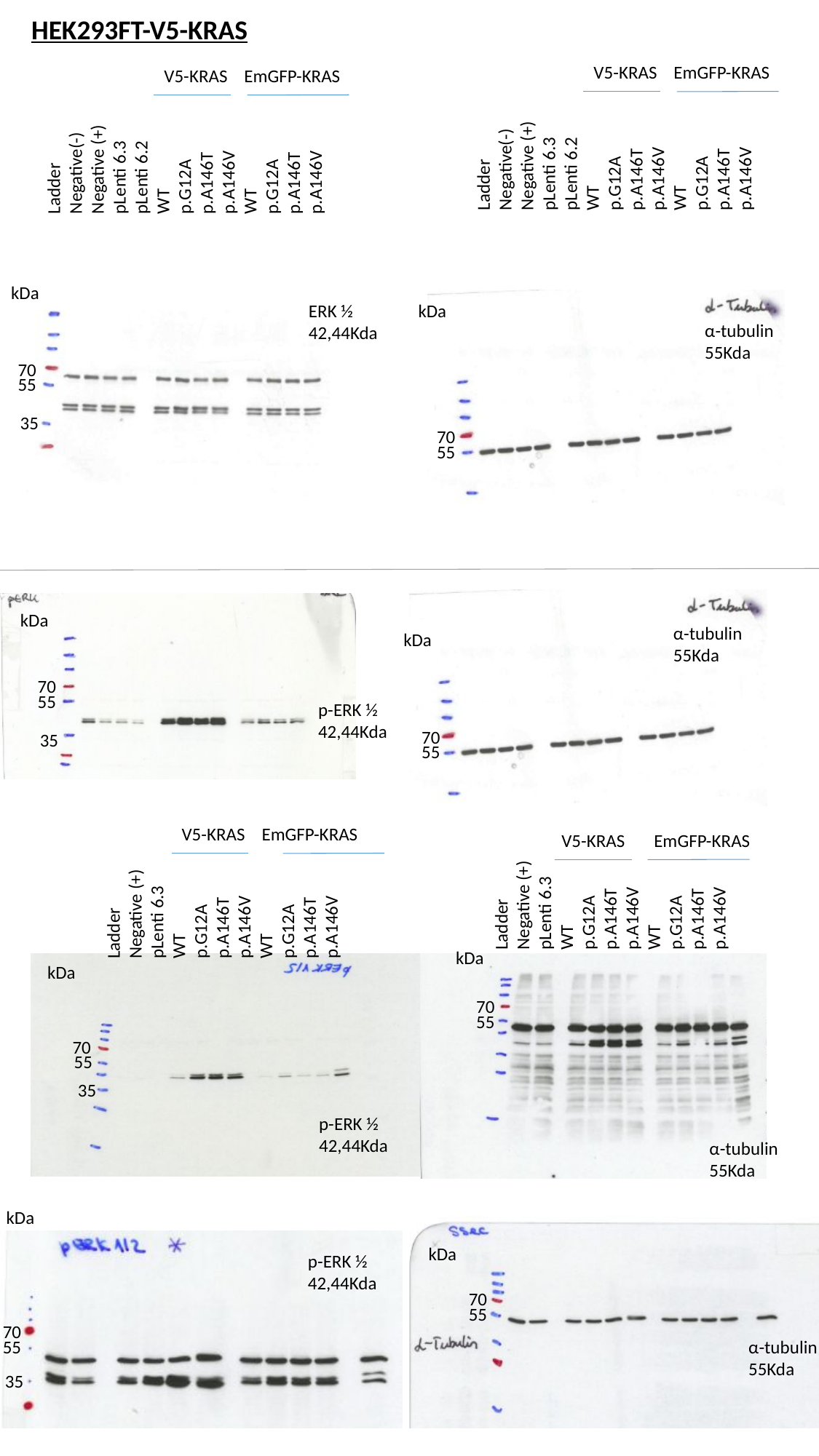

HEK293FT-V5-KRAS
Ladder
Negative(-)
Negative (+)
pLenti 6.3
pLenti 6.2
WT
p.G12A
p.A146T
p.A146V
WT
p.G12A
p.A146T
p.A146V
Ladder
Negative(-)
Negative (+)
pLenti 6.3
pLenti 6.2
WT
p.G12A
p.A146T
p.A146V
WT
p.G12A
p.A146T
p.A146V
V5-KRAS EmGFP-KRAS
V5-KRAS EmGFP-KRAS
kDa
α-tubulin
55Kda
ERK ½
42,44Kda
kDa
70
55
35
70
55
kDa
α-tubulin
55Kda
kDa
70
55
p-ERK ½
42,44Kda
70
35
55
Ladder
Negative (+)
pLenti 6.3
WT
p.G12A
p.A146T
p.A146V
WT
p.G12A
p.A146T
p.A146V
Ladder
Negative (+)
pLenti 6.3
WT
p.G12A
p.A146T
p.A146V
WT
p.G12A
p.A146T
p.A146V
V5-KRAS EmGFP-KRAS
V5-KRAS EmGFP-KRAS
kDa
kDa
70
55
70
55
35
p-ERK ½
42,44Kda
α-tubulin
55Kda
kDa
kDa
p-ERK ½
42,44Kda
70
55
70
α-tubulin
55Kda
55
35

## Slide 3
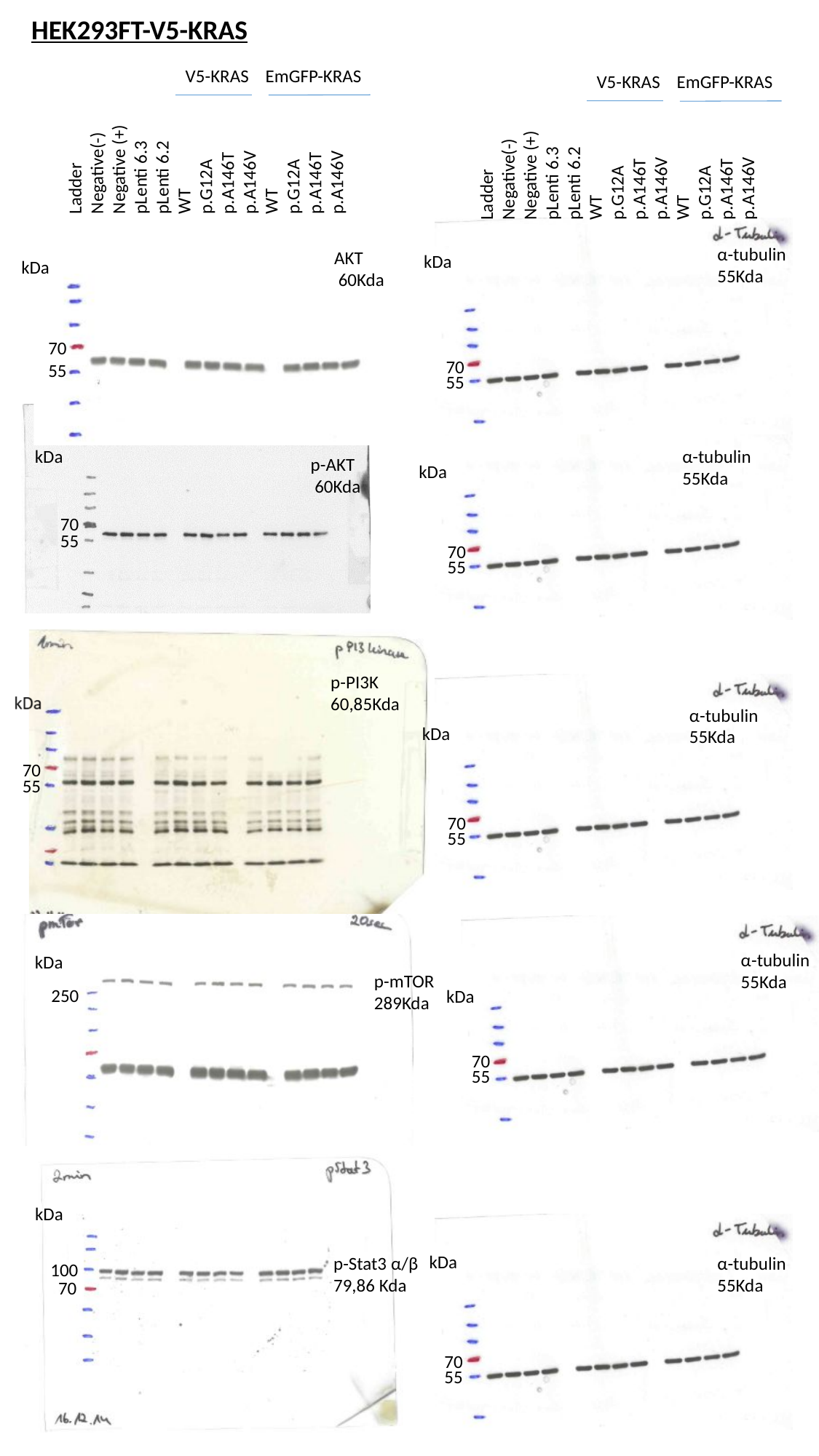

HEK293FT-V5-KRAS
Ladder
Negative(-)
Negative (+)
pLenti 6.3
pLenti 6.2
WT
p.G12A
p.A146T
p.A146V
WT
p.G12A
p.A146T
p.A146V
Ladder
Negative(-)
Negative (+)
pLenti 6.3
pLenti 6.2
WT
p.G12A
p.A146T
p.A146V
WT
p.G12A
p.A146T
p.A146V
V5-KRAS EmGFP-KRAS
V5-KRAS EmGFP-KRAS
α-tubulin
55Kda
AKT
 60Kda
kDa
kDa
70
70
55
55
α-tubulin
55Kda
p-AKT
 60Kda
kDa
kDa
70
55
70
55
p-PI3K
60,85Kda
α-tubulin
55Kda
kDa
70
55
70
55
p-mTOR
289Kda
α-tubulin
55Kda
kDa
250
kDa
70
55
p-Stat3 α/β
79,86 Kda
kDa
α-tubulin
55Kda
kDa
100
70
70
55
