# Supplementary material for: Exon-4 Mutations in KRAS Affect MEK/ERK and PI3K/AKT Signaling in Human Multiple Myeloma Cell Lines
Source: Cancers (Basel). 2020 Feb 16;12(2):455. doi: 10.3390/cancers12020455 (PMC7072554; doi:10.3390/cancers12020455)
Supplement: Supplementary file 1 [file cancers-12-00455-s001.zip › Supplementary material/Figure S4A_original western blots for Figure 5A_revised.pptx]

## Slide 1
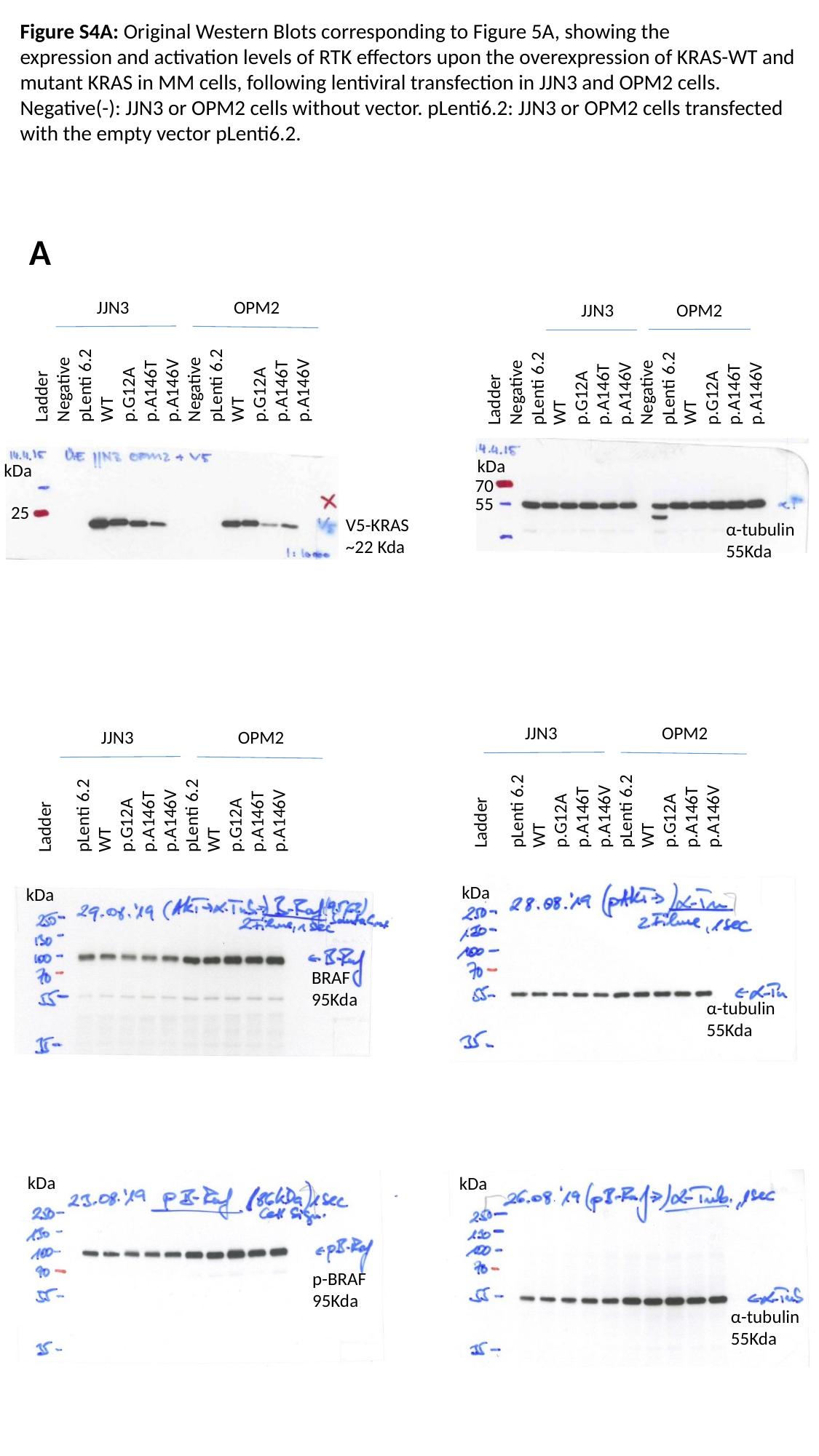

Figure S4A: Original Western Blots corresponding to Figure 5A, showing the
expression and activation levels of RTK effectors upon the overexpression of KRAS-WT and
mutant KRAS in MM cells, following lentiviral transfection in JJN3 and OPM2 cells.
Negative(-): JJN3 or OPM2 cells without vector. pLenti6.2: JJN3 or OPM2 cells transfected
with the empty vector pLenti6.2.
A
Ladder
Negative
pLenti 6.2
WT
p.G12A
p.A146T
p.A146V
Negative
pLenti 6.2
WT
p.G12A
p.A146T
p.A146V
Ladder
Negative
pLenti 6.2
WT
p.G12A
p.A146T
p.A146V
Negative
pLenti 6.2
WT
p.G12A
p.A146T
p.A146V
JJN3 OPM2
JJN3 OPM2
kDa
kDa
70
55
25
V5-KRAS
~22 Kda
α-tubulin
55Kda
Ladder
pLenti 6.2
WT
p.G12A
p.A146T
p.A146V
pLenti 6.2
WT
p.G12A
p.A146T
p.A146V
Ladder
pLenti 6.2
WT
p.G12A
p.A146T
p.A146V
pLenti 6.2
WT
p.G12A
p.A146T
p.A146V
JJN3 OPM2
JJN3 OPM2
kDa
kDa
BRAF
95Kda
α-tubulin
55Kda
kDa
kDa
p-BRAF
95Kda
α-tubulin
55Kda

## Slide 2
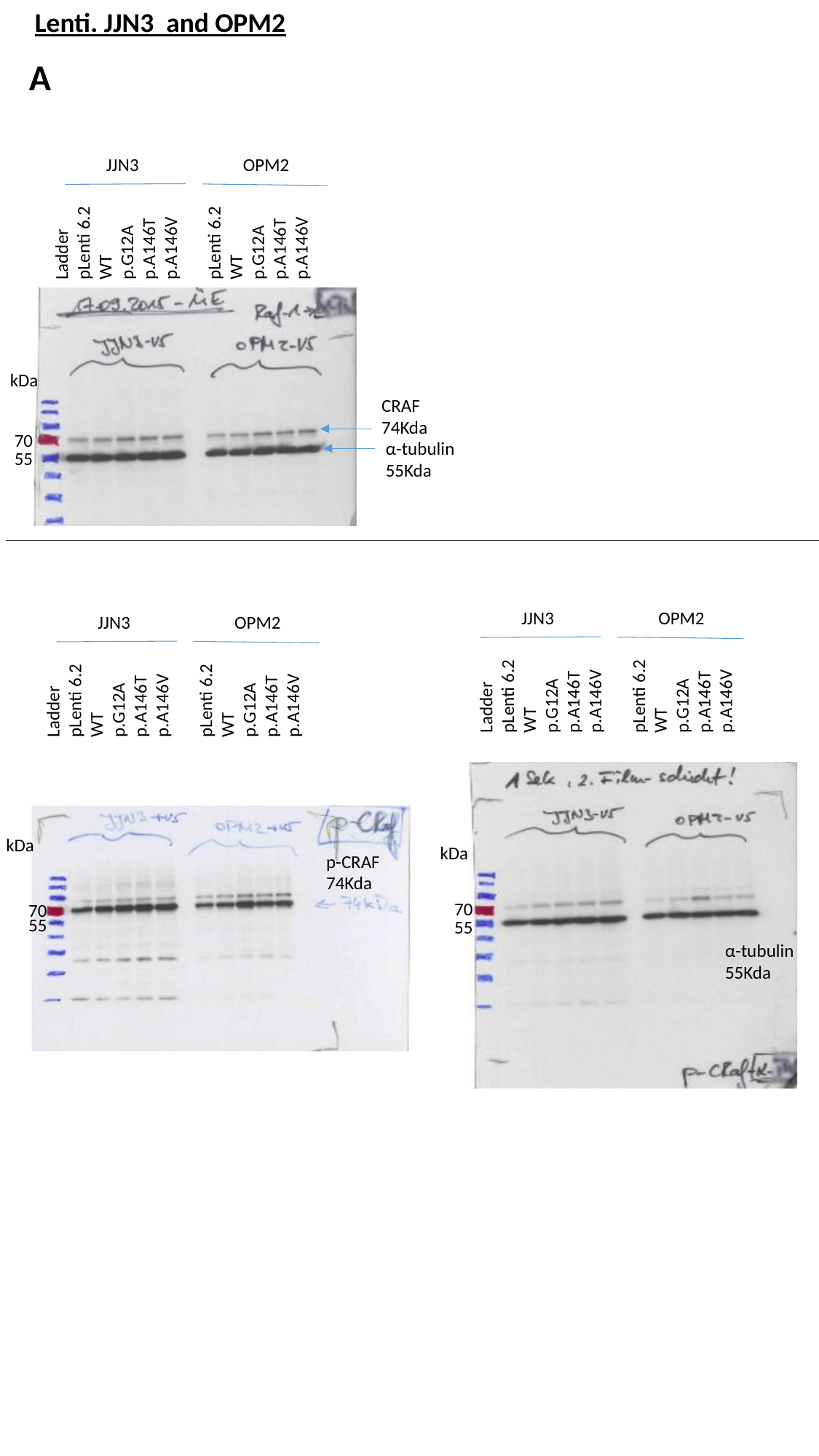

Lenti. JJN3 and OPM2
A
Ladder
pLenti 6.2
WT
p.G12A
p.A146T
p.A146V
pLenti 6.2
WT
p.G12A
p.A146T
p.A146V
JJN3 OPM2
kDa
CRAF
74Kda
70
α-tubulin
55Kda
55
Ladder
pLenti 6.2
WT
p.G12A
p.A146T
p.A146V
pLenti 6.2
WT
p.G12A
p.A146T
p.A146V
Ladder
pLenti 6.2
WT
p.G12A
p.A146T
p.A146V
pLenti 6.2
WT
p.G12A
p.A146T
p.A146V
JJN3 OPM2
JJN3 OPM2
kDa
kDa
p-CRAF
74Kda
70
70
55
55
α-tubulin
55Kda

## Slide 3
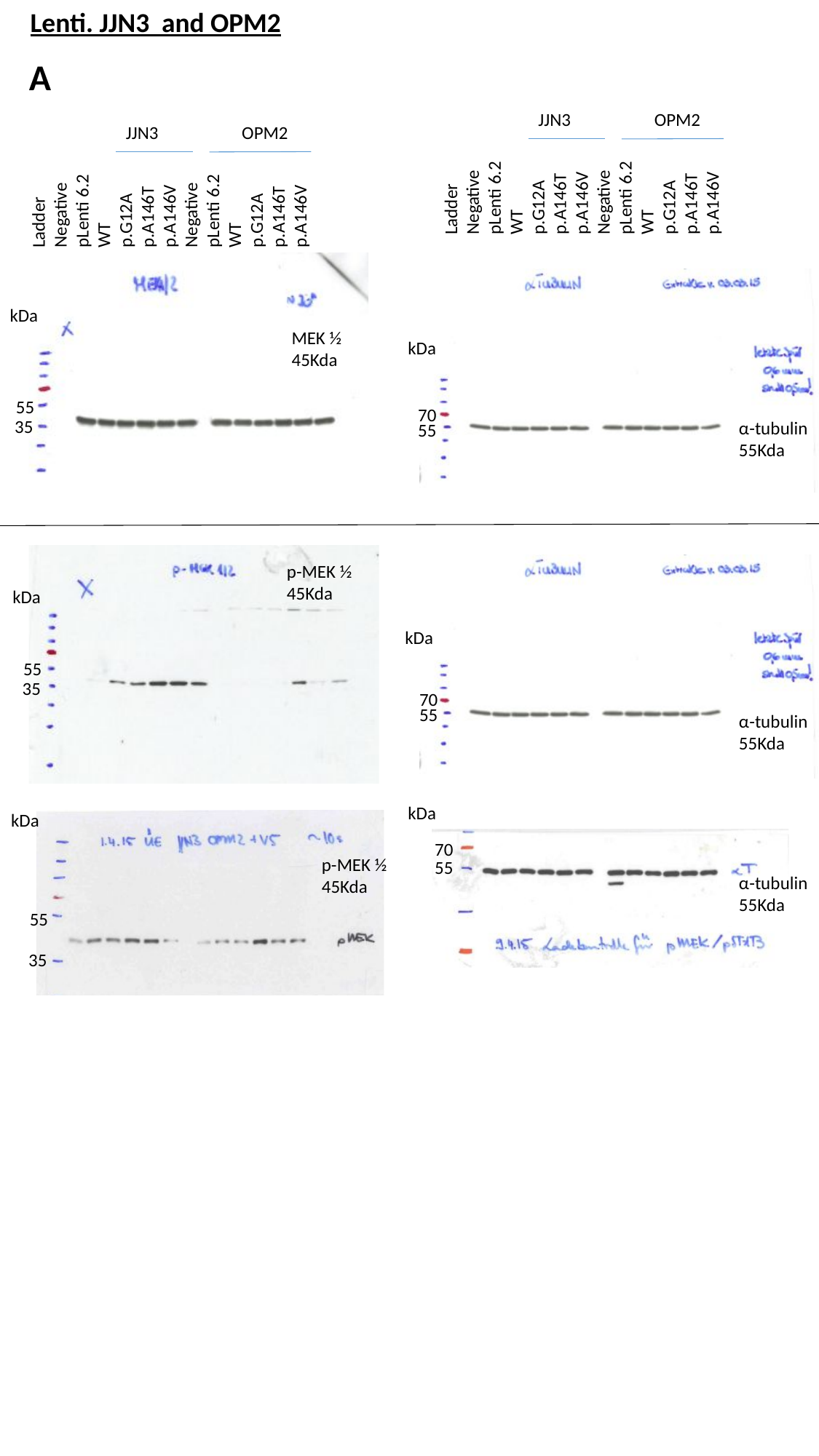

Lenti. JJN3 and OPM2
A
Ladder
Negative
pLenti 6.2
WT
p.G12A
p.A146T
p.A146V
Negative
pLenti 6.2
WT
p.G12A
p.A146T
p.A146V
Ladder
Negative
pLenti 6.2
WT
p.G12A
p.A146T
p.A146V
Negative
pLenti 6.2
WT
p.G12A
p.A146T
p.A146V
JJN3 OPM2
JJN3 OPM2
kDa
MEK ½
45Kda
kDa
55
70
35
α-tubulin
55Kda
55
p-MEK ½
45Kda
kDa
kDa
55
35
70
55
α-tubulin
55Kda
kDa
kDa
70
p-MEK ½
45Kda
55
α-tubulin
55Kda
55
35

## Slide 4
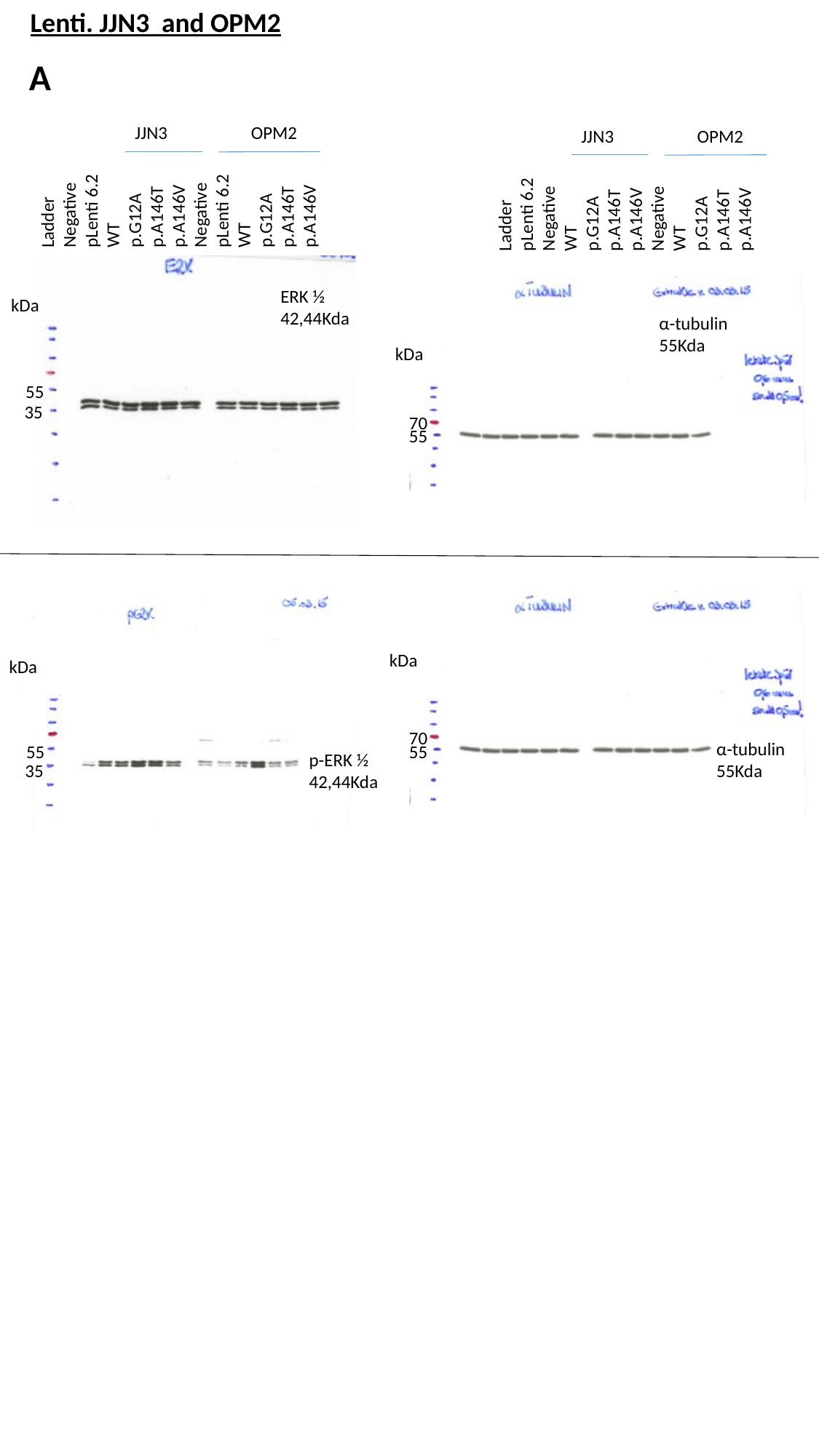

Lenti. JJN3 and OPM2
A
Ladder
Negative
pLenti 6.2
WT
p.G12A
p.A146T
p.A146V
Negative
pLenti 6.2
WT
p.G12A
p.A146T
p.A146V
Ladder
pLenti 6.2
Negative
WT
p.G12A
p.A146T
p.A146V
Negative
WT
p.G12A
p.A146T
p.A146V
JJN3 OPM2
JJN3 OPM2
ERK ½
42,44Kda
kDa
α-tubulin
55Kda
kDa
55
35
70
55
kDa
kDa
70
α-tubulin
55Kda
55
55
p-ERK ½
42,44Kda
35

## Slide 5
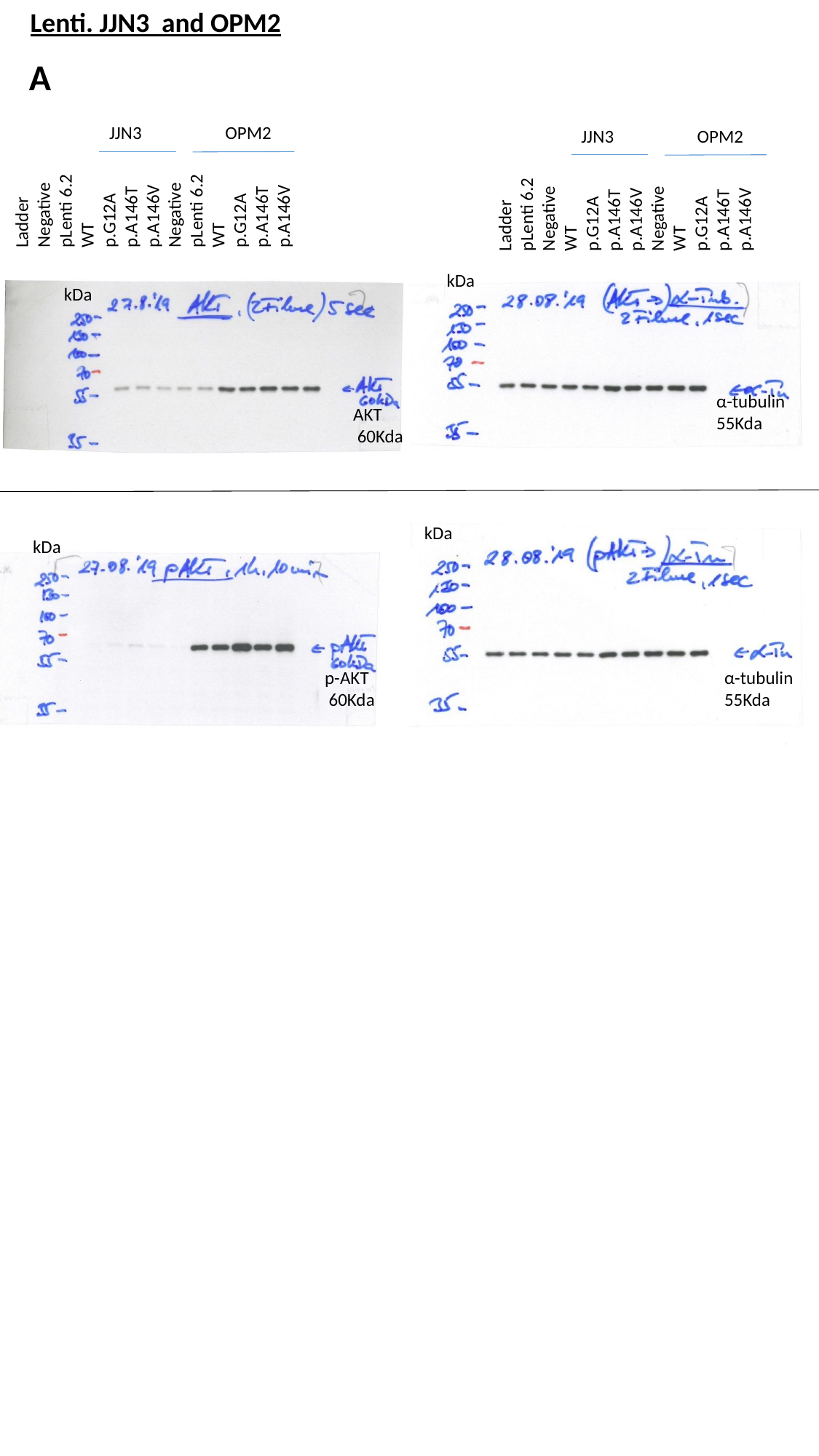

Lenti. JJN3 and OPM2
A
Ladder
Negative
pLenti 6.2
WT
p.G12A
p.A146T
p.A146V
Negative
pLenti 6.2
WT
p.G12A
p.A146T
p.A146V
Ladder
pLenti 6.2
Negative
WT
p.G12A
p.A146T
p.A146V
Negative
WT
p.G12A
p.A146T
p.A146V
JJN3 OPM2
JJN3 OPM2
kDa
kDa
α-tubulin
55Kda
AKT
 60Kda
kDa
kDa
α-tubulin
55Kda
p-AKT
 60Kda

## Slide 6
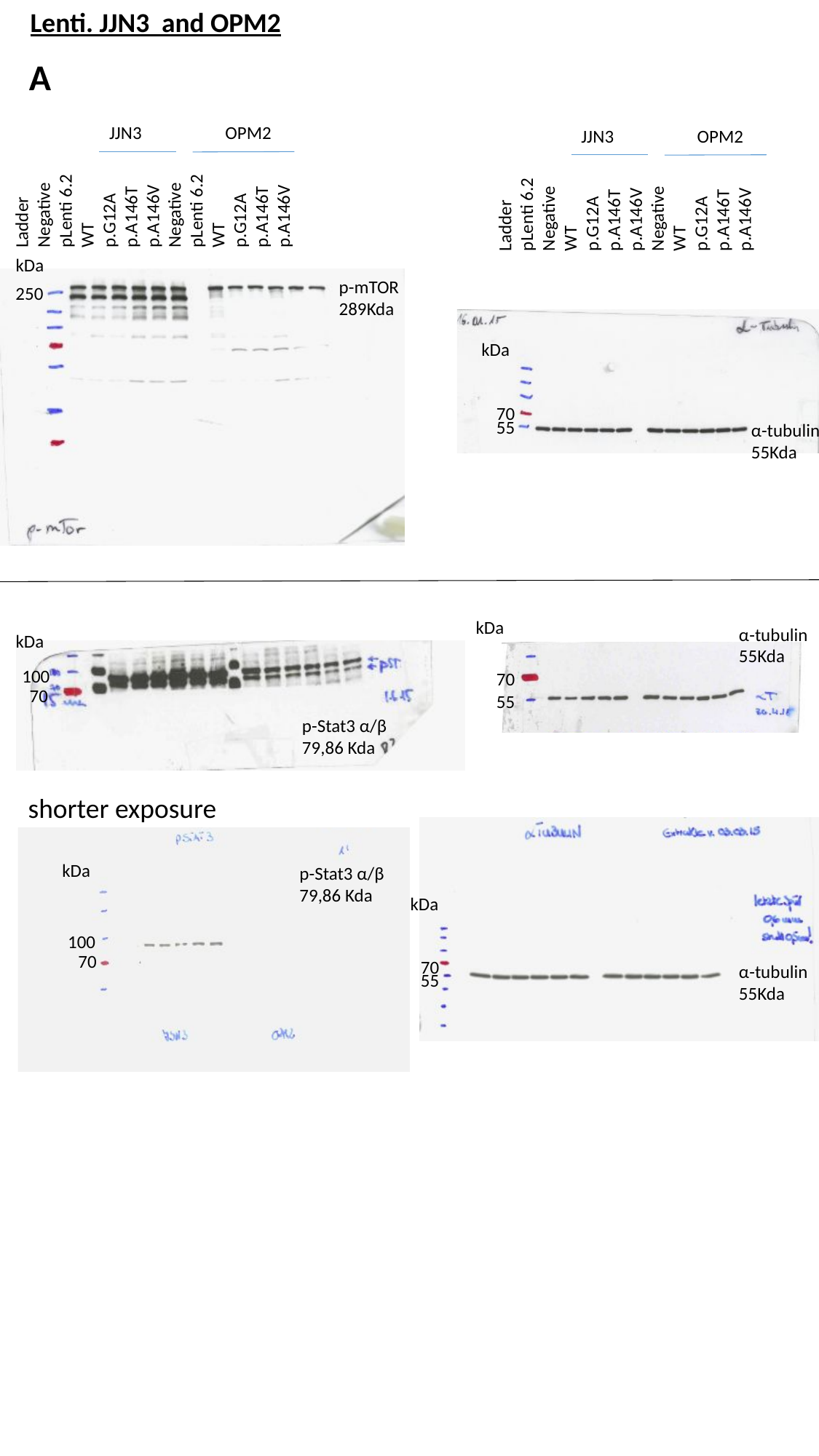

Lenti. JJN3 and OPM2
A
Ladder
Negative
pLenti 6.2
WT
p.G12A
p.A146T
p.A146V
Negative
pLenti 6.2
WT
p.G12A
p.A146T
p.A146V
Ladder
pLenti 6.2
Negative
WT
p.G12A
p.A146T
p.A146V
Negative
WT
p.G12A
p.A146T
p.A146V
JJN3 OPM2
JJN3 OPM2
kDa
p-mTOR
289Kda
250
kDa
70
55
α-tubulin
55Kda
kDa
α-tubulin
55Kda
kDa
100
70
70
55
p-Stat3 α/β
79,86 Kda
shorter exposure
kDa
p-Stat3 α/β
79,86 Kda
kDa
100
70
70
α-tubulin
55Kda
55
