# Supplementary material for: Exon-4 Mutations in KRAS Affect MEK/ERK and PI3K/AKT Signaling in Human Multiple Myeloma Cell Lines
Source: Cancers (Basel). 2020 Feb 16;12(2):455. doi: 10.3390/cancers12020455 (PMC7072554; doi:10.3390/cancers12020455)
Supplement: Supplementary file 1 [file cancers-12-00455-s001.zip › Supplementary material/Figure S4B_original western blots for Figure 5B_revised.pptx]

## Slide 1
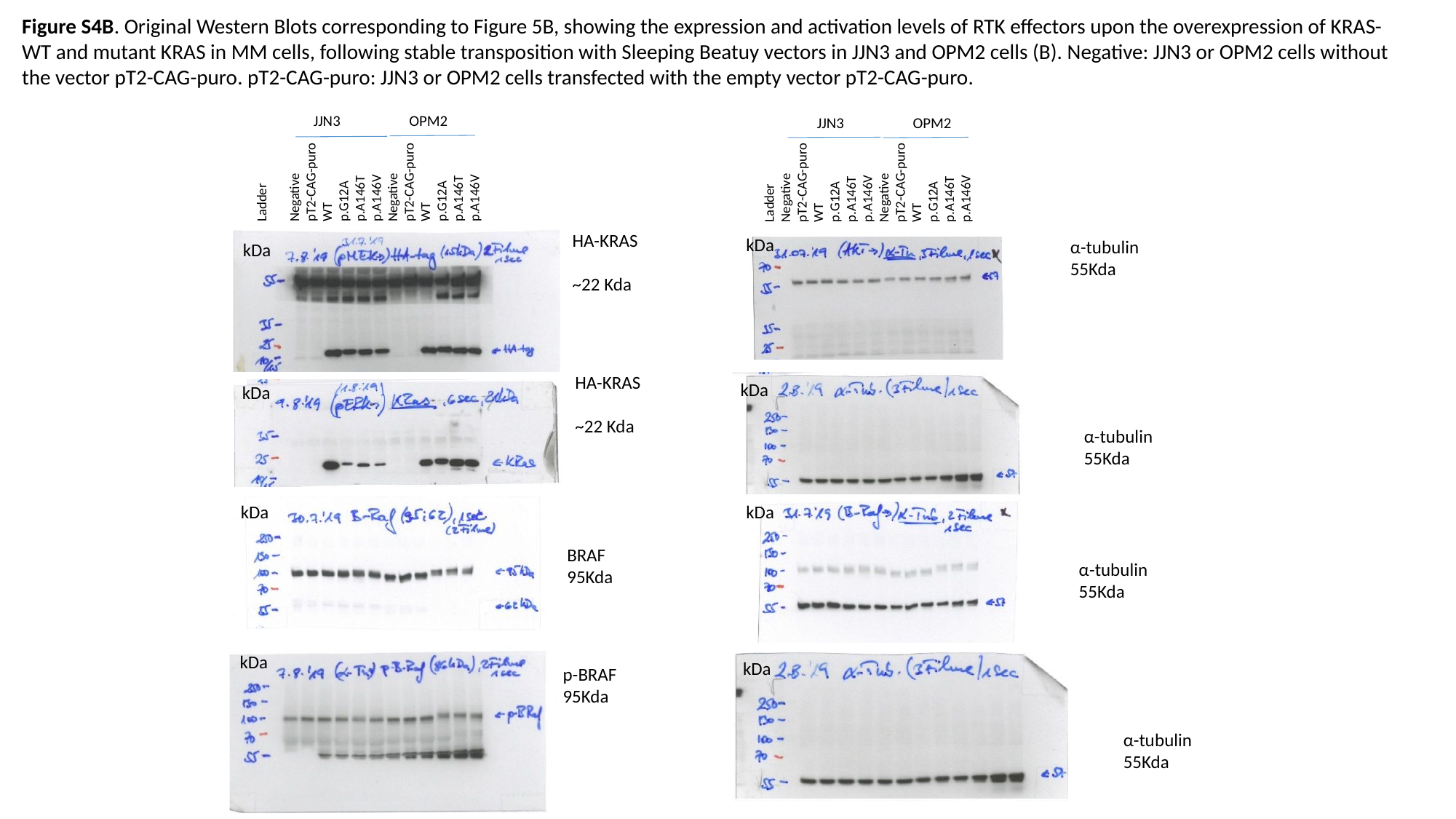

Ladder
Negative
pT2-CAG-puro
WT
p.G12A
p.A146T
p.A146V
Negative
pT2-CAG-puro
WT
p.G12A
p.A146T
p.A146V
Figure S4B. Original Western Blots corresponding to Figure 5B, showing the expression and activation levels of RTK effectors upon the overexpression of KRAS-WT and mutant KRAS in MM cells, following stable transposition with Sleeping Beatuy vectors in JJN3 and OPM2 cells (B). Negative: JJN3 or OPM2 cells without the vector pT2-CAG-puro. pT2-CAG-puro: JJN3 or OPM2 cells transfected with the empty vector pT2-CAG-puro.
Ladder
Negative
pT2-CAG-puro
WT
p.G12A
p.A146T
p.A146V
Negative
pT2-CAG-puro
WT
p.G12A
p.A146T
p.A146V
JJN3 OPM2
JJN3 OPM2
HA-KRAS
~22 Kda
kDa
α-tubulin
55Kda
kDa
HA-KRAS
~22 Kda
kDa
kDa
α-tubulin
55Kda
kDa
kDa
BRAF
95Kda
α-tubulin
55Kda
kDa
kDa
p-BRAF
95Kda
α-tubulin
55Kda

## Slide 2
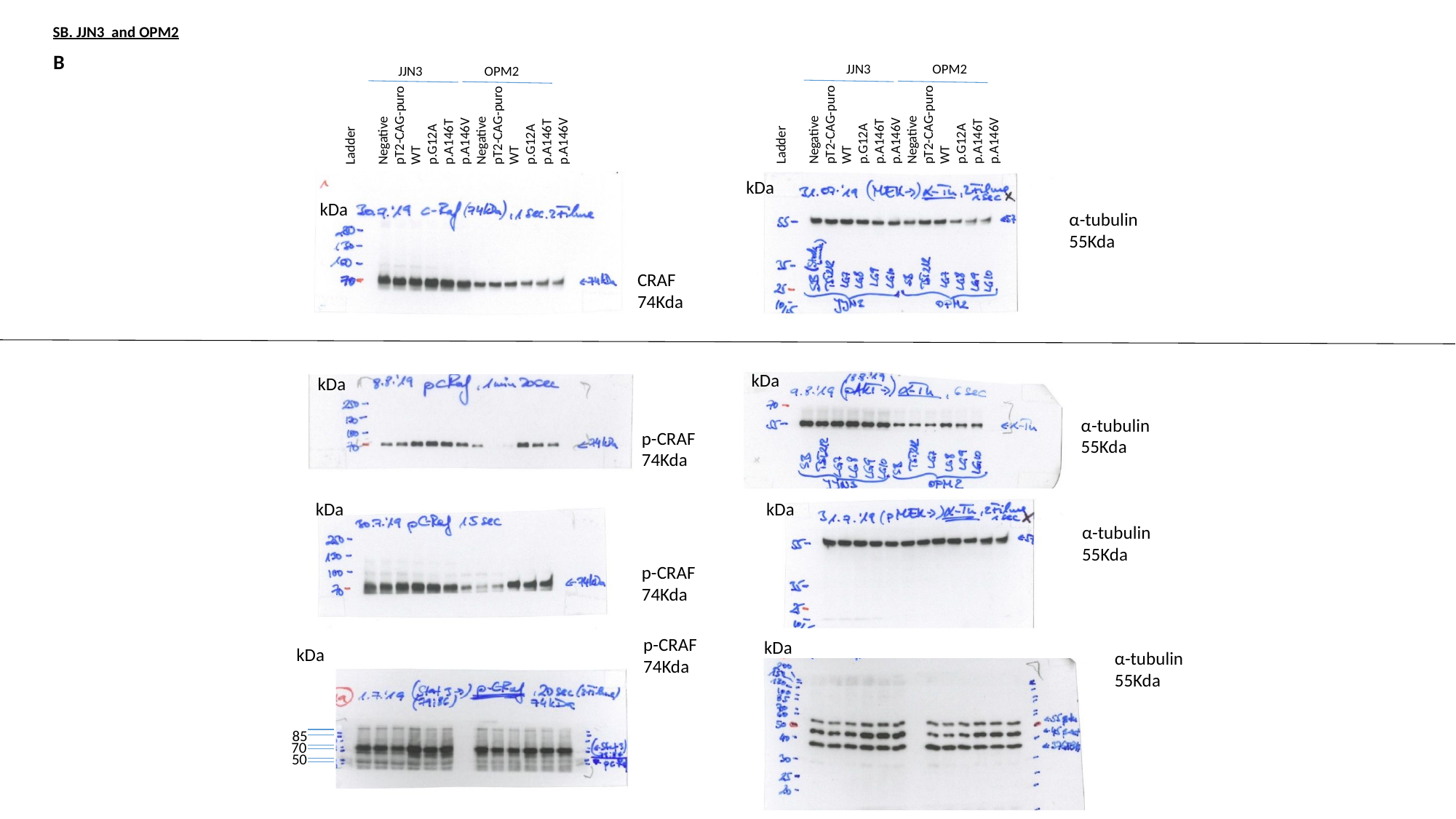

Ladder
Negative
pT2-CAG-puro
WT
p.G12A
p.A146T
p.A146V
Negative
pT2-CAG-puro
WT
p.G12A
p.A146T
p.A146V
Ladder
Negative
pT2-CAG-puro
WT
p.G12A
p.A146T
p.A146V
Negative
pT2-CAG-puro
WT
p.G12A
p.A146T
p.A146V
SB. JJN3 and OPM2
B
JJN3 OPM2
JJN3 OPM2
kDa
kDa
α-tubulin
55Kda
CRAF
74Kda
kDa
kDa
α-tubulin
55Kda
p-CRAF
74Kda
kDa
kDa
α-tubulin
55Kda
p-CRAF
74Kda
p-CRAF
74Kda
kDa
kDa
α-tubulin
55Kda
85
70
50

## Slide 3
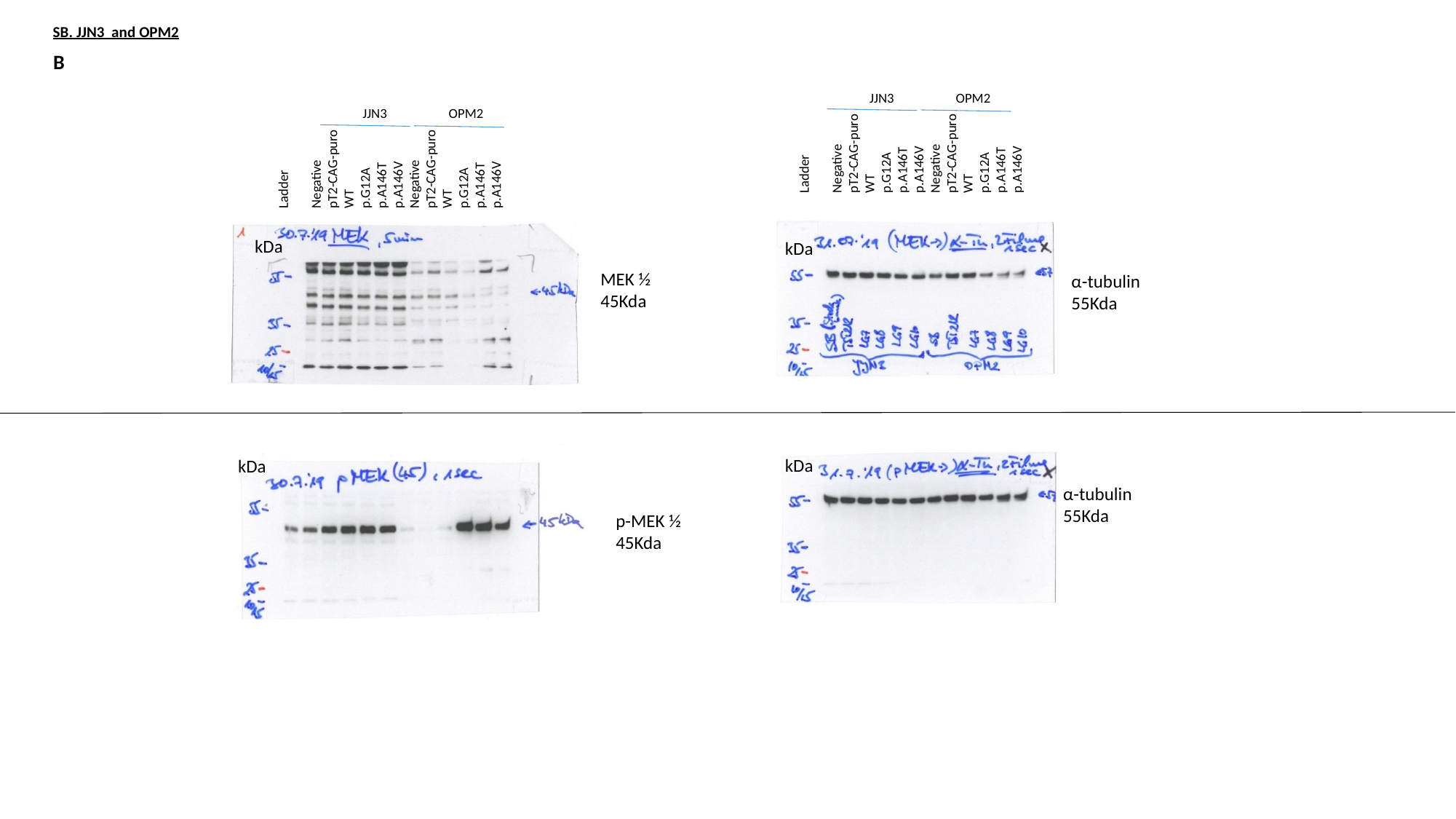

SB. JJN3 and OPM2
Ladder
Negative
pT2-CAG-puro
WT
p.G12A
p.A146T
p.A146V
Negative
pT2-CAG-puro
WT
p.G12A
p.A146T
p.A146V
B
Ladder
Negative
pT2-CAG-puro
WT
p.G12A
p.A146T
p.A146V
Negative
pT2-CAG-puro
WT
p.G12A
p.A146T
p.A146V
JJN3 OPM2
JJN3 OPM2
kDa
kDa
MEK ½
45Kda
α-tubulin
55Kda
kDa
kDa
α-tubulin
55Kda
p-MEK ½
45Kda

## Slide 4
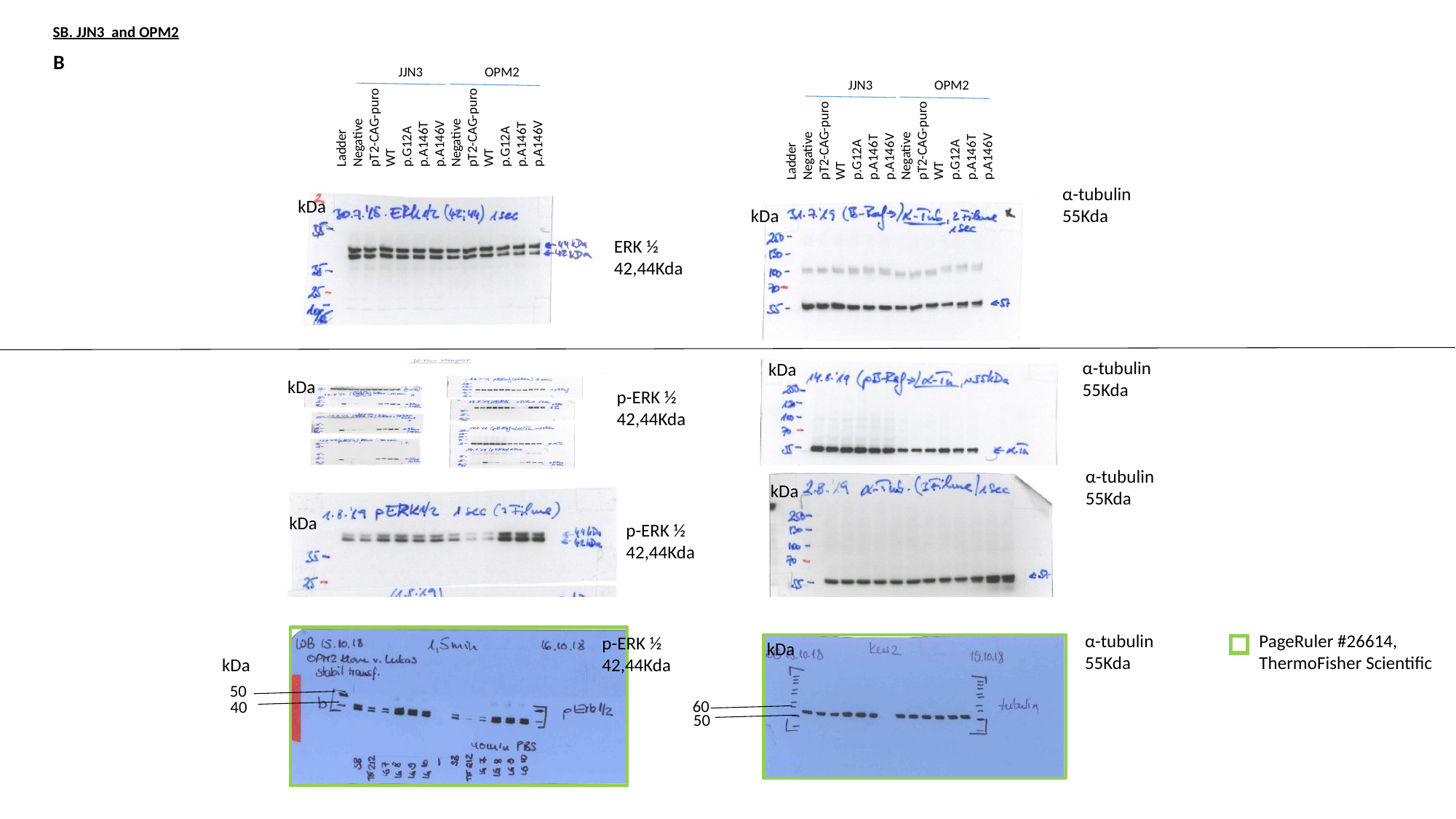

Ladder
Negative
pT2-CAG-puro
WT
p.G12A
p.A146T
p.A146V
Negative
pT2-CAG-puro
WT
p.G12A
p.A146T
p.A146V
SB. JJN3 and OPM2
Ladder
Negative
pT2-CAG-puro
WT
p.G12A
p.A146T
p.A146V
Negative
pT2-CAG-puro
WT
p.G12A
p.A146T
p.A146V
B
JJN3 OPM2
JJN3 OPM2
α-tubulin
55Kda
kDa
kDa
ERK ½
42,44Kda
α-tubulin
55Kda
kDa
kDa
p-ERK ½
42,44Kda
α-tubulin
55Kda
kDa
kDa
p-ERK ½
42,44Kda
α-tubulin
55Kda
PageRuler #26614,
ThermoFisher Scientific
p-ERK ½
42,44Kda
kDa
kDa
50
60
40
50

## Slide 5
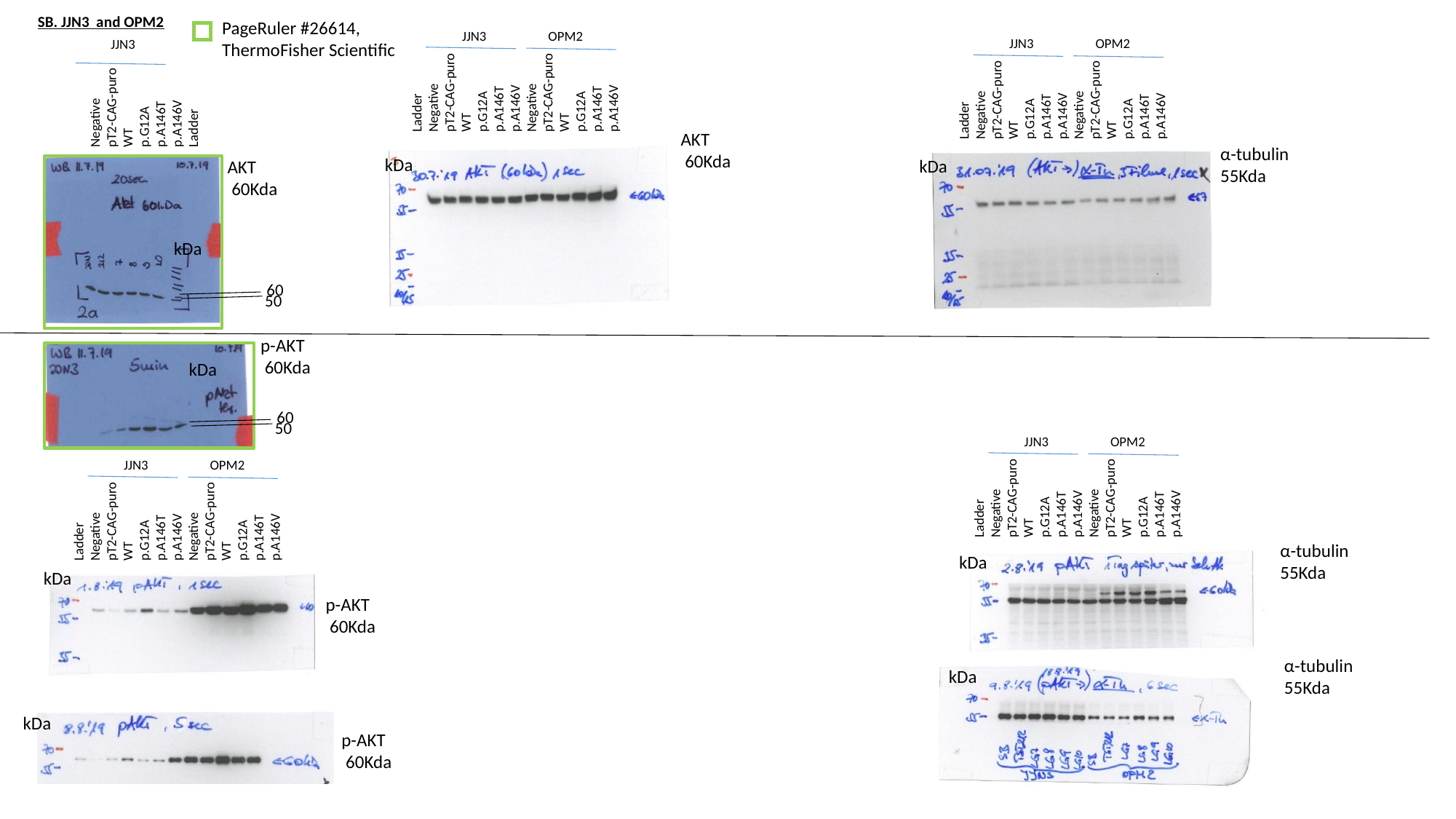

Ladder
Negative
pT2-CAG-puro
WT
p.G12A
p.A146T
p.A146V
Negative
pT2-CAG-puro
WT
p.G12A
p.A146T
p.A146V
Ladder
Negative
pT2-CAG-puro
WT
p.G12A
p.A146T
p.A146V
Negative
pT2-CAG-puro
WT
p.G12A
p.A146T
p.A146V
SB. JJN3 and OPM2
PageRuler #26614,
ThermoFisher Scientific
JJN3 OPM2
JJN3 OPM2
JJN3
Negative
pT2-CAG-puro
WT
p.G12A
p.A146T
p.A146V
Ladder
AKT
 60Kda
α-tubulin
55Kda
kDa
kDa
AKT
 60Kda
kDa
60
50
p-AKT
 60Kda
kDa
Ladder
Negative
pT2-CAG-puro
WT
p.G12A
p.A146T
p.A146V
Negative
pT2-CAG-puro
WT
p.G12A
p.A146T
p.A146V
Ladder
Negative
pT2-CAG-puro
WT
p.G12A
p.A146T
p.A146V
Negative
pT2-CAG-puro
WT
p.G12A
p.A146T
p.A146V
60
50
JJN3 OPM2
JJN3 OPM2
α-tubulin
55Kda
kDa
kDa
p-AKT
 60Kda
α-tubulin
55Kda
kDa
kDa
p-AKT
 60Kda
