# Supplementary material for: Exon-4 Mutations in KRAS Affect MEK/ERK and PI3K/AKT Signaling in Human Multiple Myeloma Cell Lines
Source: Cancers (Basel). 2020 Feb 16;12(2):455. doi: 10.3390/cancers12020455 (PMC7072554; doi:10.3390/cancers12020455)
Supplement: Supplementary file 1 [file cancers-12-00455-s001.zip › Supplementary material/Figure S4C_original western blots for Figure 5C_revised.pptx]

## Slide 1
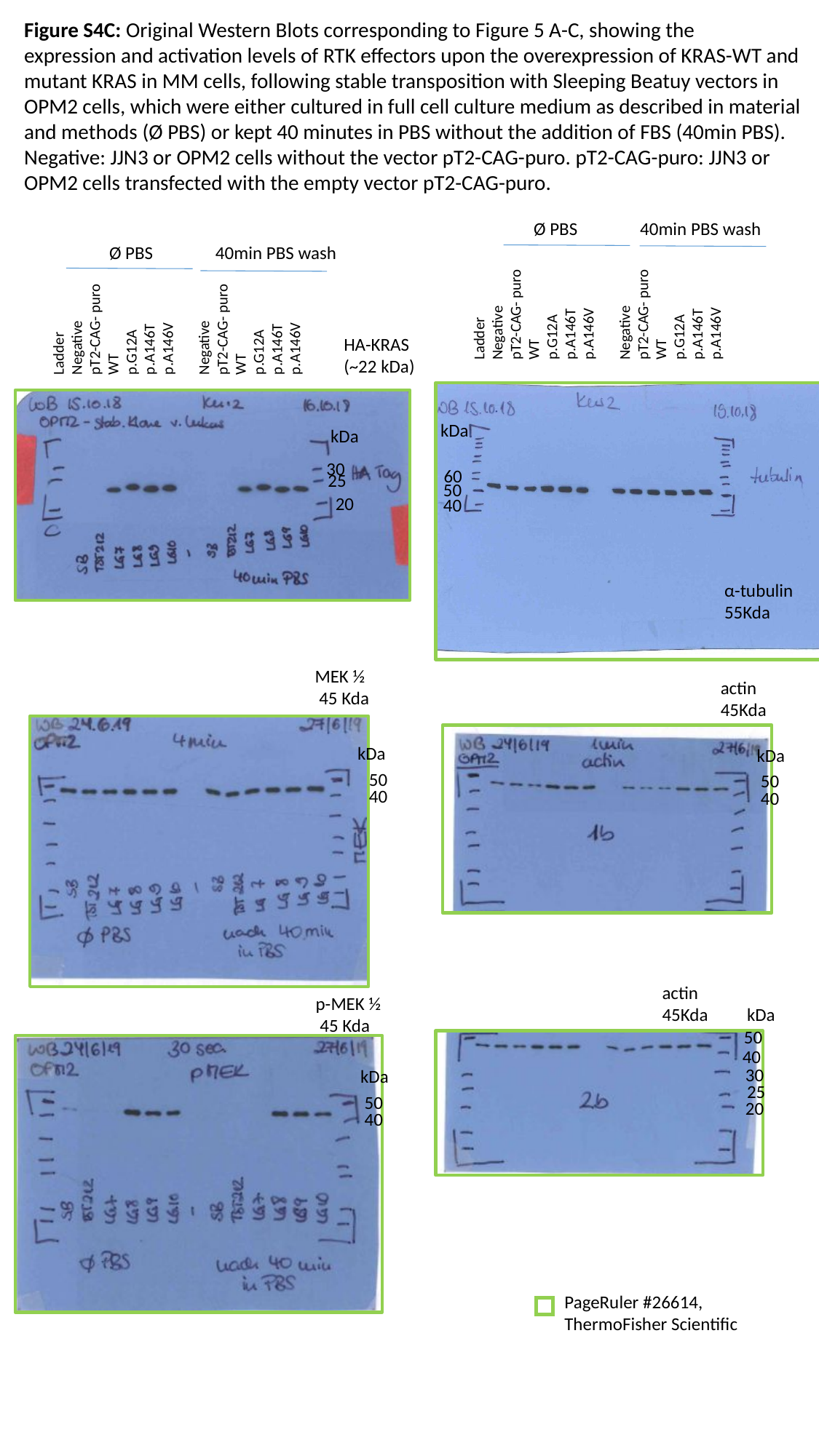

Figure S4C: Original Western Blots corresponding to Figure 5 A-C, showing the
expression and activation levels of RTK effectors upon the overexpression of KRAS-WT and
mutant KRAS in MM cells, following stable transposition with Sleeping Beatuy vectors in
OPM2 cells, which were either cultured in full cell culture medium as described in material
and methods (Ø PBS) or kept 40 minutes in PBS without the addition of FBS (40min PBS).
Negative: JJN3 or OPM2 cells without the vector pT2-CAG-puro. pT2-CAG-puro: JJN3 or
OPM2 cells transfected with the empty vector pT2-CAG-puro.
Ladder
Negative
pT2-CAG- puro
WT
p.G12A
p.A146T
p.A146V
Negative
pT2-CAG- puro
WT
p.G12A
p.A146T
p.A146V
Ladder
Negative
pT2-CAG- puro
WT
p.G12A
p.A146T
p.A146V
Negative
pT2-CAG- puro
WT
p.G12A
p.A146T
p.A146V
Ø PBS 40min PBS wash
Ø PBS 40min PBS wash
HA-KRAS
(~22 kDa)
kDa
kDa
30
60
25
50
20
40
α-tubulin
55Kda
MEK ½
 45 Kda
actin
45Kda
kDa
kDa
50
50
40
40
actin
45Kda
p-MEK ½
 45 Kda
kDa
50
40
30
kDa
25
50
20
40
PageRuler #26614,
ThermoFisher Scientific

## Slide 2
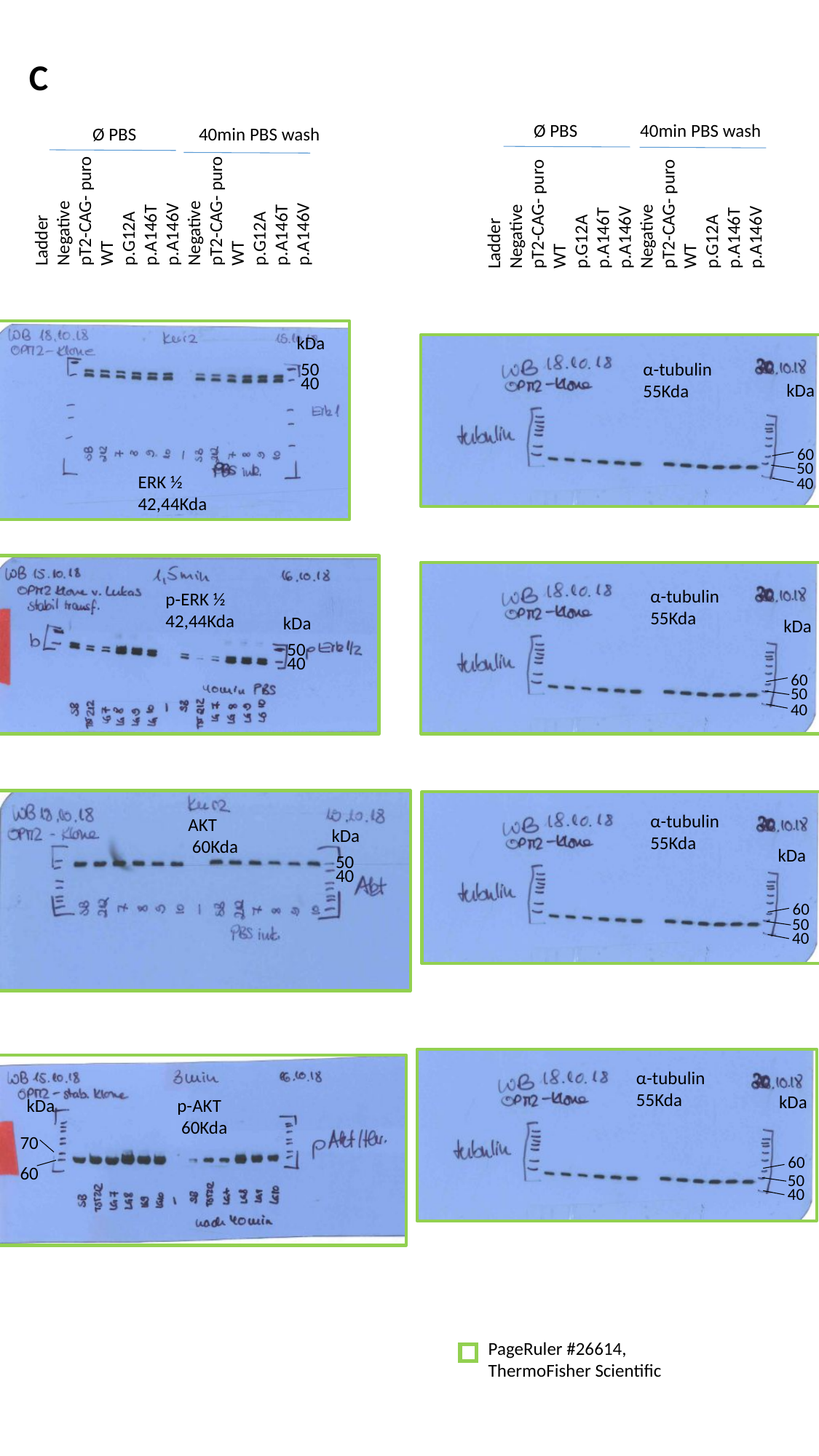

C
Ladder
Negative
pT2-CAG- puro
WT
p.G12A
p.A146T
p.A146V
Negative
pT2-CAG- puro
WT
p.G12A
p.A146T
p.A146V
Ladder
Negative
pT2-CAG- puro
WT
p.G12A
p.A146T
p.A146V
Negative
pT2-CAG- puro
WT
p.G12A
p.A146T
p.A146V
Ø PBS 40min PBS wash
Ø PBS 40min PBS wash
kDa
50
α-tubulin
55Kda
40
kDa
60
50
ERK ½
42,44Kda
40
α-tubulin
55Kda
p-ERK ½
42,44Kda
kDa
kDa
50
40
60
50
40
α-tubulin
55Kda
AKT
 60Kda
kDa
kDa
50
40
60
50
40
α-tubulin
55Kda
kDa
kDa
p-AKT
 60Kda
70
60
60
50
40
PageRuler #26614,
ThermoFisher Scientific
