# Supplementary material for: Exon-4 Mutations in KRAS Affect MEK/ERK and PI3K/AKT Signaling in Human Multiple Myeloma Cell Lines
Source: Cancers (Basel). 2020 Feb 16;12(2):455. doi: 10.3390/cancers12020455 (PMC7072554; doi:10.3390/cancers12020455)
Supplement: Supplementary file 1 [file cancers-12-00455-s001.zip › Supplementary material/Weissbach et al._Figures S1&5_AND TABLES S1, S7&S8.docx]

**SUPPLEMENTARY FIGURES AND TABLES**

Figure S1: Correlation of major and minor *KRAS* subclone mutations with cytogenetic parameters, response to therapy and survival. VAF F: variant allele frequency for the forward reads, VAF R: variant allele frequency for the reverse reads, AA: amino acid, mut: mutation


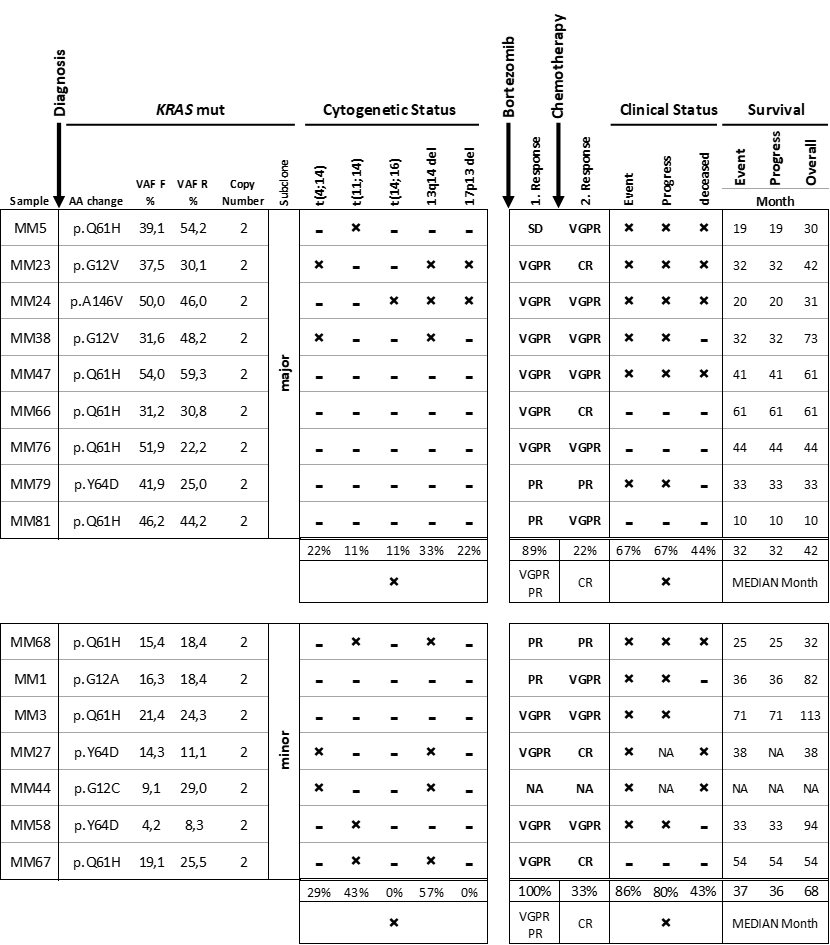


Figure S2

see separate pptx document

Figure S3

see separate pptx document

Figure S4

see separate pptx document

Figure S5: Strategy of the two-step fusion-PCR using different primer combinations to generate the fusion-proteins attB-V5-KRAS and EmGFP-KRAS (Table S III). CDS: coding sequence


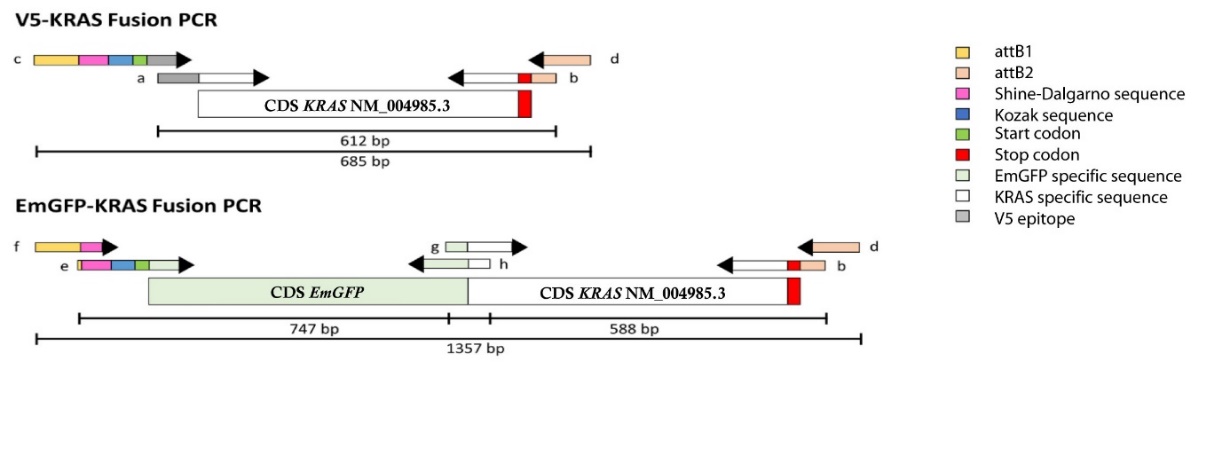


Table S1: Clinical parameters of MM patients with and without *KRAS* mutations. WT: wild type, mut: mutation, M: male, F: female, HC: heavy chain, LC: light chain, CR: complete response, PR: partial response, VGPR: very good partial response, SD: stable disease, PD: progressed disease

| **Clinical Parameters** | **KRAS mut, n=16** | **KRAS WT, n=64** | ***p*-value** | **Type of analysis** |
| --- | --- | --- | --- | --- |
| Sex; M/F | 6, 10 | 33, 30 | 0,4024 | Fisher's |
| Age at Diagnosis (years) | 53 (34-65) | 53 (32-70) | 0,9975 | Welch |
| HC; IgA, IgG, IgD, n.a. | 3, 11, 0, 2 | 14, 36, 1, 13 | 0,0938 | Two-way anova |
| LC; Lambda, Kappa | 7, 9 | 25, 39 | 0,7801 | Fisher's |
| Stage; IA, IIA, IIIA, IB, IIB, IIIB, n.a. | 2, 1, 11, 0, 0, 1, 1 | 3, 5, 39, 0, 2, 8, 7 | 0,1097 | Two-way anova |
| Response after Bortezomib: CR, PR+VGPR, SD+PD | 0, 14, 1 | 9, 41, 7 | 0,1663 | Two-way anova |
| Response after Chemotherapy: CR, PR+VGPR, SD+PD | 4, 11, 0 | 27, 18, 0 | 0,28 | Two-way anova |
| Event; no, yes | 4, 12 | 17, 45 | >0.999 | Fisher's |
| Event free survival ≥12 month; no, yes | 0, 15 | 8, 51 | 0,8921 | Log-rank |
| Progression free survival ≥12 month; no, yes | 0, 14 | 6, 48 | 0,9191 | Log-rank |
| Overall Survival ≥36 month; no, yes | 4, 11 | 18, 42 | 0,7082 | Log-rank |

Table S2

see separate excel files

Table S3

see separate excel files

Table S4

see separate excel files

Table S5

see separate excel files

Table S6

see separate excel files

Table S7: Primers for fusion PCR and for the validation of subsequent cloning procedures

| **Primer** | **Sequenz 5‘ - 3‘** |
| --- | --- |
| a | CCTATCCCTAACCCTCTCCTCGGTCTCGATTCTACGACTGAATATAAACTTGTGGTAG |
| b | GAAAGCTGGGTCTTACATAATTACACACTTTGTCTTTG |
| c | GGGGACAAGTTTGTACAAAAAAGCAGGCTTCGAAGGAGATAGAACCATGGGTAAGCCTATCCCTAAC |
| d | GGGGACCACTTTGTACAAGAAAGCTGGGT |
| e | TTCGAAGGAGATAGAACCATGGTGAGCAAGGGCGAGGAGCTGTTC |
| f | GGGGACAAGTTTGTACAAAAAAGCAGGCTTCGAAGGAGAT |
| g | TTTATATTCAGTCTTGTACAGCTCGTCCATGCCGAG |
| h | GAGCTGTACAAGACTGAATATAAACTTGTGGTAG |
| CMV F | CGCAAATGGGCGGTAGGCGTG |
| M13 F | TGTAAAACGACGGCCAGT |

Table S8: Antibodies used for Western blot and FACS analysis

| **Primary antibody** | **kDa** | **Species** | **Dilution** | **Manufacturer** |
| --- | --- | --- | --- | --- |
| HA-Tag | **-** | *Rabbit* | 1:5000 in 5% BSA/TBS-T | Abcam, Cambridge, UK |
| Anti-V5- (#13202) | - | *Rabbit* | 1:700 in 5% BSA/TBS-T | New England Biolabs, Frankfurt, Germany |
| KRAS sc-30 (#F234) | 21 | *Mouse* | 1:200 in 5% Milch/TBS-T | Santa Cruz, Dallas, USA |
| Anti-RAS | 21 | *Mouse* | 1:1000 in 5% BSA/TBS-T | Thermo Fisher Scientific, Darmstadt, Germany |
| B-Raf (55C6) | 86 | *Rabbit* | 1:1000 in 5% BSA/TBS-T | Cell Signaling Technology, Frankfurt, Germany |
| phospho-Raf-B (Thr 598/Ser 601) | 62;95 | *Goat* | 1:200 in 5% BSA/TBS-T | Santa Cruz, Heidelberg, Germany |
| phospho-Raf-B | 86 | *Rabbit* | 1:1000 in 5% BSA/TBS-T | Cell Signaling Technology, Frankfurt, Germany |
| Raf-1 (C-12) | 74 | *Rabbit* | 1:4000 in 5% BSA/TBS-T | Santa Cruz, Heidelberg, Germany |
| Phospho-c-Raf (Ser338) (56A6) | 74 | *Rabbit* | 1:2000 in 5% Milch/TBS-T | Cell Signaling Technology, Frankfurt, Germany |
| MEK1/2 (#9122) | 45 | *Rabbit* | 1:2000 in 5% Milch/TBS-T | New England Biolabs, Frankfurt, Germany |
| Phospho-MEK1/2 (S217/221)(#9154) | 45 | *Rabbit* | 1:2000 in 5% BSA/TBS-T | New England Biolabs, Frankfurt, Germany |
| p44/42 (ERK1/2) (#9102) | 42; 44 | *Rabbit* | 1:3000 in 5% BSA/TBS-T | New England Biolabs, Frankfurt, Germany |
| Phospho-p44/42- (ERK1/2)(T202/Y204)(#9101) | 42; 44 | *Rabbit* | 1:2000 in 5% BSA/TBS-T | New England Biolabs, Frankfurt, Germany |
| AKT (#4691) | 60 | *Rabbit* | 1:1000 in 5% Milch/TBS-T | New England Biolabs, Frankfurt, Germany |
| Phospho-AKT (S473) (#4060) | 60 | *Rabbit* | 1:2000 in 5% BSA/TBS-T | New England Biolabs, Frankfurt, Germany |
| Phospho-PI3K (Y458)/p55 (#4228) | 60; 85 | *Rabbit* | 1:5000 in 5% BSA/TBS-T | New England Biolabs, Frankfurt, Germany |
| Phospho-STAT3 (Tyr705) (#9138) | 79; 86 | *Mouse* | 1:500 in 5% BSA/TBS-T | New England Biolabs, Frankfurt, Germany |
| Phospho-mTOR (S2448) (#2971) | 289 | *Rabbit* | 1:1000 in 5% BSA/TBS-T | New England Biolabs, Frankfurt, Germany |
| Tubulin α AB-2 (DM1A) | 57 | *Mouse* | 1:5000 in 5% Milch/TBS-T | Neomarkers, Freemont, USA |
|  |  |  |  |  |
|  |  |  |  |  |
| Anti-V5-FITC (#R96325) | - | - | - | Life Technologies, Darmstadt, Germany |
| Annexin V APC (#31490016) | - | - | - | AL-ImmunoTools, Friesoythe, Germany |
|  |  |  |  |  |
| **Secondary antibody** |  |  | **Dilution** | **Manufacturer** |
| Polyclonal Rabbit Anti-mouse (#P0260) | - | - | 1:10000 in TBS-T | Dako, Glostrup, Dänemark |
| Anti-mouse (#7076) | - | - | 1:3000 in 5% BSA/TBS-T | New England Biolabs, Frankfurt, Germany |
| Anti-rabbit (#7074) | - | - | 1:2000 in 5% BSA/TBS-T | New England Biolabs, Frankfurt, Germany |
